# A meta-analytical review of the impact of mindfulness on creativity: Framing current lines of research and defining moderator variables

**DOI:** 10.3758/s13423-023-02327-w

**Published:** 2023-07-13

**Authors:** Zoe Hughes, Linden J. Ball, Cassandra Richardson, Jeannie Judge

**Affiliations:** 1 School of Psychology and Computer Science, University of Central Lancashire, Preston, PR1 2HE UK; 2 Department of Psychology, The University of Winchester, Winchester, SO22 4NR UK

**Keywords:** Mindfulness, Creative performance, Moderator variables, Review, Meta-analysis

## Abstract

Findings relating to the impact of mindfulness interventions on creative performance remain inconsistent, perhaps because of discrepancies between study designs, including variability in the length of mindfulness interventions, the absence of control groups or the tendencies to explore creativity as one unitary construct. To derive a clearer understanding of the impact that mindfulness interventions may exert on creative performance, two meta-analytical reviews were conducted, drawing respectively on studies using a control group design (*n* = 20) and studies using a pretest–posttest design (*n* = 17). A positive effect was identified between mindfulness and creativity, both for control group designs (*d* = 0.42, 95% CIs [0.29, 0.54]) and pretest–posttest designs (*d* = 0.59, 95% CIs [0.38, 0.81]). Subgroup analysis revealed that intervention length, creativity task (i.e., divergent vs. convergent thinking tasks) and control group type, were significant moderators for control group studies, whereas only intervention length was a significant moderator for pretest–posttest studies. Overall, the findings support the use of mindfulness as a tool to enhance creative performance, with more advantageous outcomes for convergent as opposed to divergent thinking tasks. We discuss the implications of study design and intervention length as key factors of relevance to future research aimed at advancing theoretical accounts of the relationship between mindfulness and creativity.

**Supplementary Information:**

The online version contains supplementary material available at 10.3758/s13423-023-02327-w.

Mindfulness is defined as paying attention in a particular way, on purpose, in the present moment, and nonjudgmentally (Kabat-Zinn, 1990). It has received considerable interest based on its apparently advantageous effect on cognition, which has prompted much research aimed at investigating the nature and extent of the benefits of mindfulness-based interventions on cognitive task performance. Most mindfulness-based interventions include core mindfulness techniques of focused attention and open monitoring, which aim to bring awareness to the present moment without feelings of judgment or any sense of being overwhelmed (Kabat-Zinn, 1982). Importantly, research has indicated a key role for mindfulness in enhancing performance on attentional tasks (e.g., the dichotic listening task; Chin et al., 2021), which is perhaps unsurprising given that a good degree of attentional control is required to stay engaged in mindfulness (Norris et al., 2018). Effective attentional processing is also claimed to be essential for successful performance on various high-level cognitive tasks, including creative problem-solving (Zabelina, 2011), which raises the question of whether mindfulness-based interventions can benefit creative cognition. This question forms the focus of the current review. There is agreement that mindfulness and creativity share several important processes, including attention and working memory. This has led researchers to investigate this relationship, yet the literature remains confusing and in need of simplification (cf. Lebuda et al., 2016).

The present review aims to address inconsistencies in findings using meta-analytical methods to combine independent studies and calculate the overall effects of mindfulness-based interventions on creativity tasks, while also taking into consideration the multidimensional nature of creativity. We additionally aim to address concerns that have been voiced in the literature regarding mindfulness-based interventions and methodological designs such as the type of control group present in intervention-based studies (Baer, 2003; Bishop, 2002). Although all mindfulness-based interventions share the same goal of promoting mindfulness, techniques differ considerably in terms of duration, the object of attentional focus and how attentional focus is directed toward an object. Such inconsistencies in mindfulness interventions have made it difficult to reach a consensus on the relationship between mindfulness and creativity. Before reporting our meta-analytical review, we first provide an overview of the different theoretical perspectives that have been proposed to explain the underlying mechanisms associated with mindfulness, as well as the theoretical underpinnings of the link between mindfulness and creativity.

## Theoretical perspectives on mindfulness

Mindfulness is interpreted as a set of skills that comprise both state and trait domains (de Sousa et al., 2021). Trait mindfulness refers to the innate capacity of paying and maintaining attention to the present moment with a nonjudgmental attitude, whereas state mindfulness is the intentional and deliberate practice of mindfulness, which leads to a state of awareness and sustained attention to the present moment (Tang, 2012). Without intervention, there are consistent findings that trait mindfulness is stable over time, and enhanced with regular mindfulness training (Kiken et al., 2015). Traditionally, the literature has tended to focus on *mindfulness* as a unitary construct, rather than as an umbrella term that encompasses both trait and state characteristics and various underpinning facets. However, defining such an overall mindfulness construct is difficult, as mindfulness is developed and elicited by a range of techniques that tap into different processes (Goldberg et al., 2016).

In terms of the underlying mechanisms of mindfulness, many researchers emphasize the necessity of cultivating attention regulation early in practice (Wolkin, 2015). A good degree of attentional control is required to stay engaged in mindfulness, with many researchers reporting improved attentional control as an effect of single and repeated mindfulness practice (Malinowski, 2013). Monitoring practices, like maintaining a direct focus on a chosen object (e.g., the breath) are often introduced first in mindfulness interventions, to train attention to observe present-moment experience and disengage from distractors (Lindsay et al., 2018). This may account for consistent reports of improved attentional control following both long-term (Wimmer et al., 2020) and short-term (Thompson et al., 2021) mindfulness practice.

The idea that attentional control is the primary factor in mindfulness practice is also reinforced by the fact that there is a component of attention included in most proposed models of the mechanisms of mindfulness. Shapiro et al. (2006) highlighted the importance of attention alongside attitude and intention in proposing an explanation for the beneficial effects of mindfulness on cognition. Tang et al. (2015) later claimed that attentional control and emotional regulation contribute to overall improved self-awareness, which is how mindfulness elicits beneficial effects on cognition. Importantly, too, despite the overlap between mindfulness and other modes of meditation (e.g., both require participants to sit quietly for a period), it is the specific characteristics of mindfulness that distinguishes it from meditation so as to elicit beneficial outcomes for cognition. Indeed, the same beneficial outcomes are not reported for active control group comparisons that mirror mindfulness, but which exclude nonjudgmental awareness (MacCoon et al., 2012; Noone & Hogan, 2018; Rosenkranz et al., 2019). We further note that the nonjudgmental awareness component of mindfulness is not a feature of personality constructs such conscientiousness (e.g., B. W. Roberts et al., 2014).

Considering the importance of attentional processes in mindfulness practice, it is necessary to explore the three networks of attention, namely, alerting responses, orienting to sensory stimulation, and monitoring of thoughts, actions and emotions (Giovannoli et al., 2021). The Attention Network Task (ANT; Fan et al., 2002) is an experimental task that measures the three networks of attention and that is therefore commonly used as an index to explore the effects of mindfulness on alerting, orienting, and executive control. Kwak et al. (2020) reported increased orienting and alerting using the ANT following an investigation into the effects of a 4-day mindfulness course. There are, however, some studies that report no change to attentional processes following mindfulness interventions, including long-term mindfulness-based stress reduction courses (MBSR; Anderson et al., 2007). Despite consistent reports highlighting the importance of attentional processes in the underlying mechanisms of mindfulness, the current review recognizes the inconsistencies in findings and the need for further research to understand fully the link between mindfulness and attention.

There is also growing evidence that attentional processes play a role in supporting successful working memory (WM)—that is, the brain’s capacity selectively to maintain and manipulate goal-relevant information without distraction (Oberauer, 2019). It is, therefore, unsurprising that mindfulness has also been shown to improve WM, given its strong links with attentional processes. Researchers have suggested that specific mindfulness techniques, such as focusing on a specific object (e.g., the breath), activate the same neural networks that are used for attentional and WM processes, facilitating greater efficiency (Schöne et al., 2018). Furthermore, a range of brain imaging research exists that has investigated the effects of mindfulness on WM, which generally indicates that the use of mindfulness stimulates neural regions associated with WM (e.g., the prefrontal cortex: Bailey et al., 2019; Li et al., 2021; Mrazek et al., 2012). Bailey et al. (2019) proposed that increased WM capacity in mindfulness practitioners might not be a result of higher WM activity but instead arises from the deployment of an alternative neural strategy during WM tasks, potentially allowing for more accurate responses with less neural activation. However, these results are yet to be replicated.

It is also noteworthy that the current understanding of mindfulness is starting to shift away from perceiving it as a unitary concept toward viewing it from a multicomponent perspective whereby it entails various facets. When considering the latter approach, research consistently demonstrates strong, positive correlations between trait mindfulness and performance in different cognitive control tasks (Ruocco & Direkoglu, 2013; Schmertz et al., 2009) as well as between different facets of trait mindfulness and specific aspects of cognitive function (de Sousa et al., 2021). For example, individuals with high levels of “observing”, which is a mindfulness facet describing individuals’ awareness, have better visual working-memory performance, whereas those high in “nonreactivity” (also referred to as “acceptance”) show greater cognitive control flexibility, assessed with a colour-word Stroop task (Anicha et al., 2012). These findings suggest that trait mindfulness could potentially exert a moderating influence over the effects of mindfulness interventions on cognitive function, such that those with high trait mindfulness may benefit more from such interventions. However, this is speculative as measures of trait mindfulness are not included in all intervention studies.

In comparison to trait mindfulness, state mindfulness increases during, and immediately after, mindfulness training (Tang, 2012). Heightening state mindfulness with experimentally induced practice increases trait mindfulness over time, which benefits cognition (Kiken et al., 2015). However, this fails to explain why improvements to cognition from singular, short-term mindfulness practice are reported (e.g., Thompson et al., 2021). Individual rates of change in state mindfulness over the course of interventions may be important in predicting improvements to cognitive processes, including attention. Alternatively, individual rates of change in state mindfulness may indicate individuals’ willingness to change in other adaptive ways as well, for example, through increased exercise (Masicampo & Baumeister, 2007). These changes could potentially predict changes in cognition and may explain improvements in cognition that are linked with state mindfulness. Utilizing this multicomponent perspective on mindfulness is also relevant when considering how researchers are measuring mindfulness. Widely used measures of mindfulness include the Five Facet Mindfulness Questionnaire (FFMQ; Baer et al., 2006), the Mindful Attention Awareness Scale (MAAS; Brown & Ryan, 2003), and the Kentucky Inventory of Mindfulness Scale (KIMS; Baer et al., 2004). There are, however, many concerns with employing questionnaires that evaluate self-perceptions of mindfulness, with some critiques noting the inadequate content validation of these scales as well as differences in conceptual definitions of mindfulness and the absence of any confirmation of participants’ understanding of questionnaire items (Park et al., 2013). However, the aforementioned scales show acceptable internal validity, with Cronbach alphas ranging from 0.67 to 0.93 for FFMQ, 0.78 to 0.92 for MAAS, and 0.72 to 0.97 for KIMS. The FFMQ, in particular, has high convergent validity with attention and awareness (Goldberg et al., 2016). It is also important to note that all three scales are predominantly measures of *trait* mindfulness, whereas mindfulness-based interventions heighten *state* mindfulness. As noted, regular practice may lead to changes in *trait* mindfulness, for which the FFMQ, KIMS, and MAAS are suitable measures. However, the effects of one-off interventions inducing changes in state mindfulness may not be adequately measured with the use of these scales, which has contributed to some of the inconsistent conclusions within the field.

Furthermore, Chiesa (2013) has criticized several measures of mindfulness, including the MAAS, noting that although these scales provide compelling evidence to suggest that they measure a definition of mindfulness that encompasses different perspectives and psychological theories, significant methodological deficiencies limit the possibility of drawing definitive conclusions about the specificity of these questionnaires. These deficiencies include the use of case-control designs to compare expert meditators with nonmeditator controls, the dearth of adequate control groups to which subjects are randomly assigned, and data being derived from nonclinical populations—all of which contribute to the difficulty of forming definitive conclusions about the effects of mindfulness. Self-reported trait mindfulness and experimentally induced mindfulness also elicit different outcomes (Bravo et al., 2018), again emphasizing that findings are dependent on the type of mindfulness measured.

As the field continues to grow, it becomes increasingly important to reach a consensus as to what constitutes mindfulness to enable the development of theoretical accounts. Considering the complex nature of mindfulness, and the lack of mindfulness studies adopting a multicomponent perspective, it is currently difficult to examine mindfulness as a multicomponent construct (Grossman, 2008). The differentiation of concepts involved in mindfulness remains poorly investigated and further research is required to understand mindfulness practitioners’ trajectories of state and trait mindfulness separately. As a result, we continue here to maintain the unitary construct perspective of mindfulness to ensure consistency with the studies that we have drawn upon for our reported meta-analyses.

## Theoretical perspectives on creativity

Attention and WM are important for a range of cognitive processes, including creativity. In the literature, creativity is often defined as the generation of ideas that have originality and effectiveness (Corazza, 2016; Runco & Jaeger, 2012), with originality sometimes being referred to as novelty or rarity, and with effectiveness sometimes being described as utility, fit, or usefulness. However, contemporary definitions of creativity tend to be more nuanced and sophisticated, emphasizing the importance of contextual variables, such as the social or cultural situation in which ideas, objects, or actions are generated (Plucker et al., 2004).

Such contextualized definitions are often less wedded to the importance of effectiveness in denoting an idea as original, instead considering the importance of dimensions such as meaningfulness, surprise, or aesthetic value (e.g., Acar et al., 2017; Simonton, 2017; Weisberg, 2018). For example, “everyday creativity” is often defined as the production of something original and meaningful, omitting the judgment of effectiveness, because daily creative work may not be immediately constructive but may be beneficial in the future (e.g., Goslin-Jones & Richards, 2018). Indeed, the belief that creative ideas must be effective is also questionable because it excludes the possibility that such ideas might have little use but are nevertheless “brilliant failures” (cf. Dix et al., 2006). Given the broad focus of the current research on the relation between mindfulness and creativity, we favour a more encompassing definition of creativity that is not overly constrained by a commitment to the notion of effectiveness.

At a more detailed level of analysis, a distinction exists between creative tasks that primarily involve *divergent thinking* versus creative tasks that primarily involve *convergent thinking* (Guilford, 1967). Divergent thinking tasks involve the production and assessment of multiple creative ideas over a short period of time, as arises, for example, in the Alternative Uses Task (AUT), where participants are asked to generate unusual uses for a common object such as a brick (e.g., Guilford, 1967). Convergent thinking tasks, on the other hand, involve making connections between different ideas to determine a single, correct solution to a problem, as arises, for example, in the Remote Associates Task (RAT; Mednick & Mednick, 1967), where participants are asked to find a fourth word that links three presented cue-words (e.g., “same,” “tennis,” “head”). The solution word (in the case “match”) may be associated with one or more of the cue-words through semantic association (“tennis match”), synonymy (“same” = “match”) or compounding (“matchhead”).

Although it is recognized that divergent and convergent thinking tasks are rarely “process pure” (i.e., divergent thinking tasks can involve convergent thinking and likewise convergent thinking tasks can involve divergent thinking), it is nevertheless the case that these task labels persist in the literature. Indeed, much of the research on the impact of mindfulness on creativity has explicitly used the divergent versus convergent task distinction to demarcate the focus of reported investigations. As such, for the present review we have likewise retained this task-based distinction so as both to align with extant studies in the mindfulness domain and also to enable the convergent versus divergent nature of tasks to be used as a key moderator variable in our meta-analyses that are reported below. In the following sections we detail current theorizing relating to the processes involved in divergent thinking tasks and in convergent thinking tasks, before returning to discuss the basis of the potential link between mindfulness and creativity.

### Theoretical perspectives on divergent thinking tasks

The emerging consensus in relation to people tackling divergent thinking tasks such as the AUT is that the operation of two core processes is essential, that is, the *generation* of ideas (or “ideation”) and the *evaluation* of ideas so as to retain those that are seen to have value (Smith & Ward, 2012). Interestingly, too, research with the AUT has revealed that initial answers are typically mundane and that ideas become more unusual and original over time (Christensen et al., 1957). This serial order effect in ideation means that as creativity increases over time, then the fluency of idea production reduces. To explain this, researchers typically draw on theories relating to semantic memory, in that ideas are retrieved from existing, stored knowledge (Cogdell-Brooke et al., 2020; Hass, 2017). Idea generation can assist with the production of novel ideas, whereby individuals merge two or more verbal or visual concepts, retrieved from existing, stored knowledge, that were originally completely separate (Wisniewski, 1997). Since memory retrieval differs from person to person, these findings also point toward the possibility of individual differences arising in creativity (Carlsson et al., 2000).

Idea generation has been viewed in the literature as being more closely linked to implicit, associative processing, whereas idea evaluation has been considered to be more reliant on explicit, analytic processing (e.g., R. P. Roberts & Addis, 2018; Sowden et al., 2015). This dynamic interplay between implicit ideation and explicit evaluation has led to the proposal that the way in which people tackle divergent thinking tasks can be understood from a “dual-process” perspective (e.g., Gilhooly et al., 2015), that involves them drawing upon two qualitatively distinct types of processing, termed “Type 1” and “Type 2*”* thinking (Evans & Stanovich, 2013). Type 1 processes are defined as being undemanding of WM and autonomous. Type 1 processes also tend to be fast, high capacity, nonconscious and capable of running in parallel, but these are viewed as being correlated features rather than defining features. Type 2 processes, in contrast, are defined as requiring WM and being focused on cognitive decoupling (i.e., imagining and evaluating alternative possibilities) as well as mental simulation (e.g., envisaging and assessing cause-effect relationships). Type 2 processes also tend to be slow, capacity limited, conscious and serial, but these are again considered to be correlated features rather than defining features (Evans & Stanovich, 2013).

Accounts of how people tackle divergent thinking tasks such as those advanced by R. P. Roberts and Addis (2018) and Sowden et al. (2015) emphasize the dual processes of generating and evaluating ideas, and appeal to dual-process models of cognition to capture the way in which creativity emerges out of the interplay between Type 1 associative processing and Type 2 evaluative processing. Although there are certainly exceptions to this aforementioned alignment (e.g., ideas can be generated analytically by Type 2 processing and can be evaluated intuitively by Type 1 processes), the neuroscientific evidence that derives from brain imaging research also points to this proposed alignment. For example, associative processing as can arise during idea generation has been shown to be linked to the operation of the Default Mode Network (DMN), whereas attentional processing involving WM and executive functions as can arise during idea evaluation has been linked to the operation of the Executive Control Network (ECN; Abraham, 2018; Beaty et al., 2015).

Studies of people tackling divergent thinking tasks have revealed that the DMN is engaged during resting-state processing, mind wandering, and the fluent generation of ideas (Abraham, 2018). DMN regions, however, do not seem to operate independently during divergent thinking, but instead appear to activate in conjunction or synchrony with regions associated with the ECN, which is typically linked with executive control, inhibition and WM (Beaty et al., 2015). These latter executive functions have been claimed to be especially useful for idea evaluation during divergent thinking tasks (Beaty et al., 2016; Ellamil et al., 2012) as well as for the inhibition of task-irrelevant representations (Beaty & Silvia, 2012).

To support the notion that the ECN is important for idea evaluation, a recent study by Rominger et al. (2020) observed an increase in functional coupling between idea generation and evaluation in a creative drawing task, especially in the ECN. Furthermore, participants who generated more original drawings showed greater functional connectivity across all networks that were identified in this study. Beaty et al. (2016) have suggested that specific networks forward initial ideas to the ECN for higher-order processing, like idea evaluation. In another study, Li et al. (2017) compared network transitions between low and high divergent thinking groups and reported that high divergent thinking ability was associated with more frequent transitions between different connectivity states (e.g., DMN and ECN), emphasizing the importance of cognitive switching between the DMN and the ECN for divergent thinking. In sum, the dual-process model of creativity provides an advanced understanding of the interaction between neural networks that serve to drive the generation of ideas and their subsequent evaluation, both of which are necessary for successful performance on divergent thinking tasks.

### Theoretical perspectives on convergent thinking tasks

We define convergent thinking tasks as those that primarily involve the process of connecting different ideas to determine a single, correct solution to a problem (e.g., Threadgold et al., 2018), although—as noted above—we recognize that such tasks may involve an element of divergent idea generation, especially when problems are solved via associative idea generation. One main way in which single, correct solutions come about is by means of so-called “insight,” where participants begin with a misleading or incorrect approach to a problem and need to engage in creative restructuring of the information provided to identify an effective solution (Ash & Wiley, 2006; Weisberg, 2015). Importantly, however, insights may also appear spontaneously and in the absence of an explicit problem. In these cases, impasse and restructuring stages are less salient (Stock-Homburg et al., 2021).

A key account of insight in problem-solving has been referred to as the “special-process” view (e.g., Bowden et al., 2005; Gilhooly et al., 2015)*,* because it proposes that insight is achieved through an open-minded thinking approach that is primarily driven by restructuring and associative processes that are nonconscious in nature. According to this view, attentional processes and WM are less important for insight and may even hinder the successful discovery of solutions (e.g., Ball et al., 2015; Van Stockum & DeCaro, 2020). On the other hand, the “business-as-usual” view (e.g., Ball & Stevens, 2009; Gilhooly et al., 2015) describes solutions to convergent thinking tasks as being accomplished through cognitively demanding processes, whereby participants use WM to plan and execute search strategies to reach a creative solution (e.g., MacGregor et al., 2001; Ormerod et al., 2002; Payne & Duggan, 2011). According to this view, individuals need to draw upon WM resources to represent the problem at hand, to search for new solution strategies and to keep track of already used strategies.

WM capacity varies between individuals and is often measured as the ability to focus attention on a particular task, while inhibiting distracting or irrelevant thoughts (Keller et al., 2019). Some studies have found that individuals with higher WM capacities perform better at convergent thinking tasks (Lee & Therriault, 2013; Takeuchi et al., 2020), which is evidence that appears to support a business-as-usual view of people’s processing on such tasks. Indeed, Van Stockum and DeCaro (2020) identify attentional control as being the core mechanism to explain how individual differences in WM capacity are associated with enhanced problem-solving with convergent thinking tasks, with attentional control referring to the set of processes that enable individuals to maintain goals, prioritize relevant information and avoid distractions.

The positive effect of attentional control and WM on problem-solving with convergent thinking tasks remains contested, however, as some studies have shown how situational factors that lead to increased attention can decrease effective problem-solving. For example, successful solutions appear to be hindered when participants think aloud while attempting to solve problems that are typically solved via insight processes (Ball et al., 2015; Schooler et al., 1993), supporting the special-process notion that greater attentional control can thwart insight with convergent thinking tasks.

We conclude this section by noting that neuroimaging and behavioral studies appear to support the validity of both the special-process and the business-as-usual accounts of people’s processing with convergent thinking tasks. For example, distinct patterns of neural activation (e.g., Kounios et al., 2006) and oculomotor activity (e.g., relating to pupil size and microsaccades; e.g., Salvi et al., 2020) have been observed when problems are solved via insight versus analysis. Likewise, distinct behavioural markers are linked to the sudden “Aha!” experience that occurs when a solution arises through sudden insight rather than through step-by-step analysis (Danek, 2018; Stuyck et al., 2022). This neuroimaging and behavioral evidence suggest that in multitrial studies, some convergent thinking tasks may be solved via an insight-based special process that is underpinned by nonconscious restructuring, while other tasks may be solved via an analytic, business-as-usual approach that is underpinned by a more conscious multistep process. Such evidence has contributed to the emergence of a range of “hybrid” theories of creative problem-solving with convergent thinking tasks, which recognize the existence of both special process and business-as-usual routes to solutions (e.g., Danek, 2018; Öllinger et al., 2014; Weisberg, 2015).

## Theoretical underpinnings of the mindfulness–creativity link

Following a thematic review of the available literature, Henriksen et al. (2020) claimed that there is good support for the notion that mindfulness can enhance creativity. However, there is still debate in this area and the claimed relationship seems to be contingent upon potential moderation by various contextual and problem-specific factors. Indeed, Henriksen et al. (2020) highlight several confounding variables that make it challenging to disentangle the relationship between mindfulness and creativity, including the type of mindfulness practiced (e.g., focused attention vs. open monitoring), the type of creative problems utilized (e.g., divergent vs. convergent tasks) and the multifaceted character of mindfulness itself. Here, we detail how the nature of people’s processing in relation to divergent thinking tasks and convergent thinking tasks, respectively, may account for the apparently advantageous effects of mindfulness as a unitary construct.

### Mindfulness and divergent thinking tasks

To reiterate, one of the key challenges that people face when engaging in creative thinking is how to generate new and original ideas when retrieving information from memory (Smith & Ward, 2012). Subsequent to idea generation comes the need for these ideas to be evaluated further, which manifests in a multiplicity of potential ways that work toward developing, elaborating, and enriching initial ideas (Runco, 2012). This evaluation component of creativity necessitates the inhibition of further thinking about poor ideas, as well as the selection and elaboration of good ideas.

Researchers agree that mindfulness practice can enhance many cognitive processes, including fostering greater executive control (e.g., attention, WM and emotion regulation). The neuroscientific study of mindfulness outcomes has been influential in providing evidence to support this view. For example, Taren et al. (2017) reported increased functional connectivity amongst neural regions associated with executive functions (see also Doll et al., 2015; Taren et al., 2017). The explicit, attentional processing that arises during idea generation and idea evaluation, which is required for divergent thinking, is commonly linked to the ECN. Mindfulness may enhance the executive aspects of attentional processing with tasks such as the AUT, by increasing the efficiency of these specific neural networks, thereby enhancing creativity in terms of increased creative idea generation and evaluation. In a similar vein, long-term practice of mindfulness is also associated with differences in ECN activity (Brewer et al., 2011), perhaps due to the importance of attentional control during practice (e.g., giving rise to increased sustained attention). Cognitive switching between the ECN for idea evaluation and the DMN for idea generation, allows for enhanced divergent thinking ability (Beaty et al., 2016, 2018), further highlighting the key role of the ECN, at least in respect to divergent thinking.

### Mindfulness and convergent thinking tasks

It seems likely that attentional control and WM capacity can be enhanced by mindfulness practice through activation of the same underlying brain regions, which allows them to work more efficiently (Bailey et al., 2019). Mindfulness also supports the ability to focus attention, potentially by increasing WM capacity. In light of evidence that WM and sustained attention are associated with successful performance on convergent thinking tasks, including problems that can be solved via insight, it makes sense that individuals should show better problem-solving performance on such tasks following a mindfulness intervention. This prediction was supported by Ostafin and Kassman (2012), who made use of a mix of classic insight and noninsight problems from Schooler et al. (1993), which all required convergent solutions.

Although this latter evidence aligns with the business-as-usual view of performance on convergent thinking tasks (e.g., Ball & Stevens, 2009; Gilhooly et al., 2015), directly contrary predictions would arise from the special-process view (e.g., Ball et al., 2015; Bowden et al., 2005; Gilhooly et al., 2015), given the assumption that increased attention toward a problem-solving task might be expected to hinder the operation of nonconscious processes that are essential for the insightful restructuring of given information. The special-process view could explain why some researchers (e.g., Capurso et al., 2014; DeCaro et al., 2016) report that higher WM capacity and mindfulness practice *hinder* insight in problem-solving, since both of these constructs are linked to enhanced attentional control. In sum, whether improved attentional control and higher WM capacity arising from mindfulness practice are predicted to help or hinder creative problem-solving with convergent thinking tasks is likely to depend critically on the theoretical stance that is taken.

## Empirical support for the mindfulness–creativity link

Colzato et al. (2012) partially dissected the complexity associated with the measurement of creativity by investigating the impact of mindfulness upon both convergent and divergent thinking tasks, using the RAT and AUT, respectively. Data demonstrated that performance on convergent thinking tasks increased following focused-attention practice, and that individuals performed better on divergent thinking tasks following open-monitoring practice (Colzato et al., 2012), thereby identifying a key mindfulness–creativity connection and also supporting the business-as-usual theory of insight problem-solving.

It is possible that different types of mindfulness meditation (focused-attention vs. open-monitoring) prime cognitive-control states that are needed to perform effectively on convergent or divergent thinking tasks. This hypothesis was supported by Colzato et al. (2012), yet it remains speculative until supported with additional evidence. Since Colzato et al.’s (2012) study, little further research has been conducted to explore these task-specific relationships in depth, therefore, there is still uncertainty surrounding the effects that mindfulness and its sub-facets have on creativity with convergent and divergent thinking tasks.

It is also of interest that other outcomes of practicing mindfulness have been identified in the literature, which may be important for creative problem-solving, including reduced mind wandering (i.e., attention lapses) during tasks (Rahl et al., 2017). Henriksen et al. (2020) explored the relationship between mindfulness, mind wandering and creativity and concluded that despite what appear to be opposing dynamics, mind wandering and mindfulness can simultaneously enhance creativity. This has been termed “mindful mind wandering” by Preiss and Cosmelli (2017), which nurtures creativity and differs with respect to other types of mind wandering, which are considered less useful, such as mind wandering that is unintentional. The key notion here is that mindfulness may allow for “intentional” mind wandering that facilitates more awareness so as to benefit and individual’s creative imagination (Preiss & Cosmelli, 2017).

It is noteworthy, too, that recent criticisms of creativity research (e.g., Zhang et al., 2019) highlight how studies have often failed to measure the creativity that arises on particular tasks in terms of both its convergent and divergent component processes. Indeed, as we alluded to earlier, recent evidence suggests that problems such as Compound Remote Associate Tasks (CRATs, which are versions of the RAT where solution words form compounds with each of the prime words) involve convergent thinking to select a final solution but may also rely initially on divergent thinking processes such as associative word generation (see also Marsh et al., 2021). It is additionally common for rebus puzzles, which involve combinations of visual, spatial, verbal, and numerical cues from which a participant must identify a common phrase or saying, to require both divergent and convergent creative processes (Threadgold et al., 2018). Moreover, despite historical claims that convergent and divergent thinking are separate constructs that are uncorrelated (e.g., Chermahini & Hommel, 2011), more recent research by Webb et al. (2017) has reported a small relationship (*r* = .25) between performance on problem-solving tasks that can be solved via insight processes and performance on divergent thinking tasks. In addition, Webb et al. (2017) have demonstrated a small to medium relationship (*r* = .37) between performance on tasks that can be solved via insight processes and performance on reasoning tasks designed to measure convergent thinking (for related evidence, see Shen et al., 2018).

Notwithstanding the theoretical and empirical complexities in assessing the advantages of mindfulness practice for creative performance, a wide body of research, some of which we have noted already, does appear to attest to such benefits on a range of creativity tasks, including problems that can be solved by insight. Ostafin and Kassman (2012) provided evidence that mindfulness reduces tendencies to rely on habitual responses when searching for solutions to problems that can be solved via insight, thereby improving creative performance with short-term interventions (i.e., under 1 week; Jedrczak et al., 1986). Ren et al. (2011) similarly showed that maintaining a mindful and aware state resulted in more solutions to convergent thinking tasks that can be solved via insight in comparison to an active control group. The findings reported by Ostafin and Kassman (2012) and Ren et al. (2011) therefore suggest that the attentional and WM benefits that are afforded by mindfulness can facilitate creative performance with convergent thinking tasks, which are results that are more in line with the business-as-usual view of problem-solving.

It is important to note, however, that Ren et al. (2011) did not explore divergent thinking tasks such that their results may not generalize to other types of creative thinking beyond convergent thinking tasks that can be solved by insight. Nevertheless, other research that has utilized divergent thinking tasks appears to support a mindfulness–creativity link. For example, Ding et al. (2015b) assessed creativity using the Torrance Test of Creative Thinking (TTCT; Torrance, 1972), which includes AUTs and picture completion tasks. Participants in a mindfulness group outperformed a relaxation training control group on the TTCT, highlighting how characteristics that are specific to mindfulness practice such as attentional focus are likely to be important for creative processes. However, there were substantial interindividual differences in the intervention group scores, as some participants improved greatly, some only slightly and others showed worse performance. Differences in participants’ personalities and mood states prior to mindfulness practice were hypothesized to explain differences in creativity scores (Ding et al., 2015b), pointing to the potential influence of dispositional and affective moderator variables. Based on previous findings (e.g., Carlsson et al., 2000) it is also possible that individual differences in WM capacity could have led to the discrepant findings across individuals. It is additionally worth noting that no baseline group comparisons were made in this study, such that it is possible that group differences in mindfulness traits existed prior to the study.

In contrast to these aforementioned findings, several studies have produced evidence of mindfulness practice leading to poorer creative performance in a range of creative tasks (e.g., Binder et al., 1999; Smallwood & Schooler, 2006), with these negative effects being argued to arise from a reduced ability for participants to think “intuitively” in a more nonconscious, associative, and open-ended manner (Baird et al., 2012). Zedelius and Schooler (2015) further proposed that the link between mindfulness and creativity may be dependent on how problems are approached (i.e., via nonconscious insight processes or via conscious analysis processes), after they found a negative relationship (*β* = −0.25) between mindfulness and problem-solving. Further examination revealed that decrements in successful problem-solving performance were present for problems that were approached with an insight strategy, whereas problems that were approached with an analytic strategy were not affected by mindfulness practice (Zedelius & Schooler, 2015). These latter findings are in line with the special-process view of problem-solving with convergent thinking tasks in that the enhanced attentional control and WM efficiency arising as a result of mindfulness practice hinders creativity by focusing effort on misleading problem representations and incorrect strategies (cf. Ball et al., 2015), whereas the solution to these problems requires a more attentionally diffuse, associative, and open-ended process. Given that Zedelius and Schooler’s (2015) findings were based on self-reported dispositional mindfulness, it is unclear whether experimentally induced mindfulness would produce similar effects for creative problems depending on whether they are solved via insight or via analysis.

Despite the seemingly complex nature of the relationship between mindfulness and creativity, it does appear that they share important underpinning processes, albeit with efforts to understand these common processes still being far from conclusive. To bring clarity to these issues, Lebuda et al. (2016) conducted a meta-analytical review to explore the mindfulness–creativity link, which included 20 independent samples. They found a small to medium correlation between mindfulness and creativity (*r* = .22) yet reported more between-study variation than within-study variation, suggesting that moderators may exist between studies rather than within them. In their meta-analytical review, the moderator variables that Lebuda et al. (2016) included were study design (correlational vs. experimental), the type of creativity measured (divergent vs. convergent thinking tasks) and the type of mindfulness measured (attention focused mindfulness vs. state mindfulness vs. trait mindfulness). They emphasized that because of the paucity of literature in this area, it was impossible to investigate all of the theoretically relevant moderators (e.g., intervention length) that appear to influence the impact of mindfulness on a range of factors (cf. Howarth et al., 2019). Study design revealed no effects, suggesting that the small to medium effect of mindfulness on creativity was not moderated by the design of the studies. The effect of mindfulness on creativity also tended to be stronger when the creativity measurement included convergent thinking tasks rather than divergent thinking tasks, perhaps supporting the business-as-usual view that performance on convergent thinking tasks can be enhanced through improved attentional control and available WM capacity. The relationship between mindfulness and creativity was also significantly lower when studies included the awareness aspects of mindfulness (measured using the MAAS), which can be contrasted with disinhibition and mind wandering that have previously been reported to be linked positively with creativity (Mooneyham & Schooler, 2013; Zedelius & Schooler, 2015). This latter finding sheds light on important theoretical questions concerning the nature of the association between mindfulness and creativity, as disinhibition and mind wandering are reduced by mindfulness (Xu et al., 2017).

## Aims of the present meta-analytical review

At the time of writing, the review by Lebuda et al. (2016) is the most recent meta-analytical review of the mindfulness–creativity link. The current analysis aims to offer a more up-to-date meta-analytical review of the literature than that reported by Lebuda et al. (2016), while also addressing limitations that they identify with their own approach. First, although Lebuda et al. (2016) coded “type of creativity” as a moderator variable, they dichotomized this in terms of whether the measure of creative performance was either objective or subjective in nature. Consequently, this was later emphasized as a limitation, with recommendations for future research to, “differentiate between various forms of creativity as related to mindfulness” (Lebuda et al., 2016, p. 4). Hence, the current meta-analysis aimed to include a clear distinction between convergent and divergent thinking tasks to provide a better understanding of the creativity-mindfulness link, given that these two types of creative tasks are likely to be underpinned by distinct processes that may be affected differently by mindfulness.

Lebuda et al.’s (2016) meta-analysis was further limited by the small number of moderator variables explored, which is admittedly an unfortunate consequence of a scarce literature. Their meta-analysis also lacked a focus on intervention lengths or intensities, and the present analysis aimed to address this issue by exploring intervention durations. Finally, Lebuda et al. (2016) focused on dispositional measures of mindfulness. As previously mentioned, self-reported dispositional mindfulness and experimentally induced mindfulness have the potential to elicit different outcomes (Bravo et al., 2018). The current analyses therefore focused on experimentally induced mindfulness to address this gap in the literature.

Other than the meta-analysis reported by Lebuda et al. (2016), there is an absence of other meta-analytical reviews to examine the mindfulness–creativity link. Like Lebuda et al. (2016), we acknowledge the inconsistencies that prevail across the mindfulness and creativity fields. The current review addresses the limitations of Lebuda et al. (2016) to allow us to further understand the mindfulness–creativity link and includes specific moderator constructs not fully explored previously.

To establish the efficacy of randomized controlled trials (RCTs) examining mindfulness-based interventions, Dunning et al. (2019) reviewed studies that employed RCTs with an active control condition, which refers to the use of an alternative task that may be expected to benefit participants and that also matches mindfulness interventions in terms of nonspecific factors such as the duration of the intervention. Importantly, the active control task should not include mindfulness to ensure group differences can be attributed to the absence or presence of mindfulness (see MacCoon et al., 2012, for a discussion). Only 17 appropriate studies were selected for the RCT category, highlighting the paucity of good quality research in this area.

Both Dunning et al. (2019), and McCarney et al. (2007) emphasize the efficacy of the gold standard RCT methodology, reporting positive effects of mindfulness on executive functions and attention across RCT studies only. However, as only relatively few mindfulness studies have adopted an RCT design, this means that current reviews of the mindfulness literature are confined to a small number of high-quality studies, making it difficult to be confident about the robustness of some of the findings arising from these analyses. The dearth of high-quality research also makes it difficult to reach agreement concerning certain relationships, one of which is the link between mindfulness and creativity, which forms the key focus of the present review, with existing studies having produced contradictory evidence. As we have noted, some researchers report improvements following mindfulness practice on a range of convergent and divergent thinking tasks, including the AUT and RAT (Baas et al., 2014; Ostafin & Kassman, 2012), whilst others report little to no effect (Remmers et al., 2015). Most research investigating mindfulness meditation uses complex programmes, such as MBSR. However, because these programmes include yoga, stretching, and different types of meditation, it is challenging to say whether reported improvements are the result of a particular mindfulness practice (e.g., object focus) or a combination of techniques (e.g., breathing, relaxation). This highlights the importance of accurately designed active controls to pinpoint what is causing the beneficial effects.

The methodological quality of mindfulness studies remains problematic due to small sample sizes and the lack of actively controlled longitudinal studies. Many experiments are not derived from a theoretical foundation (for a review, see Matko & Sedlmeier, 2019), meaning that conclusions are often drawn from post hoc interpretations and are therefore tentative. Despite ongoing efforts to understand the nature and benefits of mindfulness interventions, there remains uncertainty towards how mindfulness affects other constructs, like creativity. Criticisms of mindfulness research are common in most reviews (e.g., Lebuda et al., 2016) with methodological and conceptual problems being at the core of much of this critique. Most evaluations of mindfulness-based interventions involve either uncontrolled or nonrandomized trials, which can limit conclusions.

Bishop (2002) was one of the first to highlight the methodological limitations that pervade the mindfulness literature, noting a host of concerns, including the lack of active control groups and a preponderance of repeated-measures designs. Similarly, Baer (2003) also mirrored these concerns, specifically lamenting the use of nonactive controls, noting that although treatment-as-usual control groups account for change due to time, an active control is needed to account for the influence of demand characteristics and placebo effects.

Another control-group approach that is utilized in many mindfulness studies is to include a waiting-list control group, which involves participants who receive the same intervention as those in the experimental group but at a later time-point. There are, however, concerns that employing waiting-list control designs may artificially inflate intervention effect estimates (Cunningham et al., 2013). This concern was later confirmed by Furukawa et al. (2014) following a meta-analysis comparing different types of control groups. As expected, waiting-list controls were found to exaggerate the efficacy of a cognitive behavioural therapy intervention (Furukawa et al., 2014). Individuals placed in waiting-list control groups may also already have interests and engage with mindfulness practice ahead of the intervention, which can give rise to inconclusive findings regarding the efficacy of mindfulness-based interventions. Another issue with waiting-list control groups is that participants are rarely monitored during the waiting time and researchers are unaware of any other lifestyle changes or interventions that participants may seek during this time that may influence findings. Researchers therefore recommend the use of active control groups in lieu of waiting-list control groups, which encompass fewer methodological issues (Park et al., 2013).

In summary, there are multiple underpinning cognitive processes that are important for both mindfulness and creativity, yet to date there is no consensus as to the effects that mindfulness practices have in terms of enhancing or disrupting creative performance. It is therefore timely to undertake a review of the existing literature on mindfulness interventions and creativity to determine the extent to which mindfulness has an impact on creativity and to identify any moderator variables that have an influence on the mindfulness–creativity link. The current meta-analytical review draws together the available evidence from empirical studies of mindfulness and creativity to identify key findings and provide a directional steer to help guide future research in this area. The more detailed objectives of this review can be summarized in the form of two specific research questions (RQs):

- RQ1: Is there reliable evidence that mindfulness practice can influence creativity, either positively or negatively?
- RQ2: What are the most influential moderators that impact upon any observed link between mindfulness and creativity?

## Overview of the meta-analyses

To address the frequent absence of control groups in existing studies (Farias & Wikholm, 2016; Goyal et al., 2014; MacCoon et al., 2012) the decision was taken to conduct two separate meta-analyses, with each centred on one of the two main types of study design reported in the literature. The first meta-analysis therefore focuses on mindfulness and creativity studies that include control group designs, whereas the second focuses on mindfulness studies that involve a pretest–posttest design. This approach allows for conclusions to be drawn from studies utilizing these distinct design features, potentially further underscoring the importance of control group designs in advancing an overall consensus in the mindfulness literature. Both meta-analyses that are reported included key moderator variables as appropriate to the study design being focused on. These moderator variables are described in the next sections.

### Moderator 1: Type of control group

The first meta-analysis that is reported related to studies that included control groups. To reiterate, a key methodological limitation of current research that has employed control group designs relates to the type of control group that is selected. The presence of a control group in intervention-based studies has been shown to be influential to the outcome of the study (Goyal et al., 2014; MacCoon et al., 2012). Waiting-list control groups appear to be less useful than more valid comparison conditions, such as active control groups (Hesser et al., 2011), which is a criticism that also extends to poorly chosen active control conditions, where overlap of characteristics being investigated exist between experimental and control conditions (Farias & Wikholm, 2016). Only 3% of published meditation intervention studies included active control treatments in 2014 (Goyal et al., 2014) and even nowadays most studies investigating mindfulness interventions still lack any form of control group. It is, therefore, important to consider the type of control group as a moderator variable in the first analysis, and to assess how findings relating to control group designs vary from those implementing pretest–posttest designs.

### Moderator 2: Convergent and divergent creative tasks

Research has indicated the importance of considering different types of creative tasks when examining the relationship between mindfulness and creative performance (e.g., Ding et al., 2014; Jedrczak et al., 1986; Zedelius & Schooler, 2015). From a theoretical standpoint, it therefore seems important to draw a distinction between convergent and divergent thinking tasks for the present meta-analyses and to include such tasks as a moderator variable. This approach affords an opportunity to provide a more refined assessment of the impact of mindfulness on creativity to advance theoretical understanding, providing clarification of the discrepant results noted earlier, and thereby guiding future research.

### Moderator 3: Mindfulness intervention length

The benefit of mindfulness interventions on cognition (e.g., sustained attention) is currently contested in terms of the length and intensity of such interventions (Mrazek et al., 2012). Long-term, daily practice over a course of several months certainly seems to be capable of improving attentional processes (Giannandrea et al., 2019), including intensive practice arising in the context of an 8-week MBSR course (Josefsson et al., 2011; see also Kabat-Zinn, 2003). Recent research has also provided evidence for mindfulness practice improving sustained attention regardless of the length of practice, implying that even short interventions can effectively stimulate attentional processes (Mrazek et al., 2012). Indeed, Larson et al. (2013) showed improvements to sustained attention after short, low-intensity interventions (i.e., 10–20 min daily for 1 week), with evidence for enhanced error awareness and mitigated posterror slowing (i.e., reduction in reaction times following an error trial) during a flanker task. Likewise, specific enhancements to creative performance have been observed with short mindfulness interventions of under one week (Jedrczak et al., 1986). In sum, the possibility that intervention length plays an important role in the effects of mindfulness practice on creativity lends weight to its inclusion as a moderator variable in the present meta-analyses.

## The meta-analyses

### Literature search strategy

Publications were identified that contained studies relevant to a meta-analysis of mindfulness and creativity through a search of PubMed, Google Scholar, Ovid, Scopus, Science Direct, and Cochrane Library databases, using the keywords mindfulness, and creativ*, intersected with one of convergent, divergent, intervention, or problem. Unpublished work was also identified using ResearchGate and employing the same aforementioned keywords. Where necessary, authors were contacted for any supporting documents, which allowed for the computation of effect sizes. Authors cited in the reference lists of published articles were also contacted when searching for unpublished work. A total of 37 studies are included in the current review, following a strict inclusion/exclusion process (see Appendix A of the Supplemental Material for a summary of the inclusion criteria; see Appendix B of the Supplemental Material for the PRISMA flowchart; Haddaway et al., 2022). Studies matching the criteria for both analyses, for example, including a combination of pretest–posttest and control group designs, were included in both meta-analyses (*N* = 10; Baas et al., 2014; Ding et al., 2014, 2015a, b, Gouda et al., 2016; Justo et al, 2014; Müller et al., 2016; Ostafin & Kassman, 2012; Poure, 2016; Ren et al., 2011; Walsh, 2013).

### Transparency and openness

We adhered to the Meta-Analytic Reporting Standards (MARS) for meta-analytic reporting (Appelbaum et al., 2018). All meta-analytic data and research materials have been made publicly available at The Open Science Framework (https://osf.io/e9ums/?view_only=9d8dadd4017e4a4c9b392539dda473af).

Data were analyzed using Comprehensive Meta-Analysis (CMA; Bornstein et al., 2013) Version 3.0. This review project was not preregistered.

### Addressing publication bias

Publication bias (also referred to as reporting bias) is a well-recognized issue in meta-analyses and relates to the absence of information caused by either the nonpublication of entire studies or the selective outcome reporting in studies based on their results (Harbord et al., 2006). Studies with statistically significant findings (i.e., *p* < .05) are also more likely to be published, and published sooner, compared with nonsignificant studies (*p* > .05; for a review see Duyx et al., 2017). Publication bias is especially problematic in RCTs, since it leads to inflated and unreliable results regarding different treatments or interventions (Rothstein et al., 2006). Identification and control of publication bias is therefore essential to preserve the validity of meta-analytical reviews. To help ameliorate publication bias in the current literature search, we included doctoral dissertations, unpublished articles and conference articles, which were located using Google Scholar and ResearchGate.

Various methods are available to test the extent and impact of publication bias in meta-analyses. Our approach involved calculating the standard error and standard difference in means across conditions to create funnel plots to assess the potential role of publication bias in both analyses (Harbord et al., 2006; see Appendix C and D of the Supplemental Material). In the absence of publication bias, we would expect studies to distribute symmetrically around the combined effect size. Duval and Tweedie’s trim and fill test, Begg and Mazumdar rank correlation test, and Egger’s test of the intercept were also used to assess publication bias in both samples. To support further the robustness of our publication-bias analyses, we also conducted classic fail-safe* N* and Orwin’s fail-safe *N* analyses to address the possibility that studies were missing from the analysis and, if included in the analysis, would impact the overall effect size. We implemented the same scoring method used in Ribeiro et al. (2016) to categorize bias as being: (1) high, if three of the publication-bias tests indicated bias; (2) moderate, if two of the tests indicated bias; and (3) low, if just one test indicated bias (see Appendix H and I of the Supplemental Material for the publication-bias analyses).

#### Assessing publication bias in studies utilizing control groups

Observational analyses revealed symmetry in the funnel plot for studies utilizing control group designs, with Duval and Tweedie’s trim and fill analysis finding no evidence of missing effect sizes. Fail-safe *N* analysis indicated that the weighted mean effect size for this sample was a robust nonzero effect. Begg and Mazumdar’s rank correlation test and an Egger’s test of the intercept both determined no publication bias in this sample. We conclude that the sample of studies utilizing control group designs displays no degree of publication bias.

#### Assessing publication bias in studies utilizing pretest–posttest designs

For studies using pretest–posttest designs, observational analysis revealed asymmetry in the funnel plot, which is indicative of publication bias. Further observation of the funnel plot revealed a relatively high number of small studies falling toward the right of the mean and we express concerns that studies falling to the left of the mean may exist but may be missing from this analysis. However, Duval and Tweedie’s trim and fill analysis concluded that there were no missing effect sizes. Fail-safe *N* analyses indicated that the weighted mean effect size for this sample was a robust nonzero effect. Begg and Mazumdar’s rank correlation test suggested significant publication bias (see Table 1); however, an Egger’s test of the intercept determined no publication bias in this sample.

**Table 1:** Publication bias by outcome across control group and pretest–posttest studies

Test of publication bias	Control group studies	Pretest–posttest studies
Classic Fail-Safe *N*	431 effect sizes	68 effect sizes
Orwin’s Fail-Safe *N*	65 effect sizes	50 effect sizes
Begg & Mazumdar’s Rank Correlation Test	*B* = .01,* p* = .97	*B* = 0.14, *p* < .05
Egger’s Test of the Intercept	*B* (18) = −.00, *p* = .99	*B* (15) = −.05, *p* = .90
Duval and Tweedie’s Trim and Fill Method	0 effect sizes missing	0 effect sizes missing
Overall Degree of Publication Bias	None	Low

Even though observational analyses revealed some asymmetry in funnel plots, five tests of publication bias reported either no publication bias or low degrees of publication bias. Arguably, such tests provide more accurate interpretations of publication bias, since the visual examination is subjective (Lin & Chu, 2018). For this reason, we conclude that the sample of studies utilizing pretest–posttest designs displays a low degree of publication bias, basing this decision on the outcome of the five tests as detailed in Table 1.

### Quality assessment of studies included in both meta-analyses

Although there are no objective criteria for assessing study quality in the literature, we sought to consider the quality of the studies identified in our meta-analyses and identified nine key factors that are important and relevant to empirical studies utilizing mindfulness interventions: (1) the quality of statistical reporting; (2) details about the mindfulness intervention employed; (3) the appropriateness of statistical tests; (4) baseline group differences; (5) participant withdrawal rates; (6) randomization methods; (7) mindfulness measures; (8) the clarity of research questions/aims; and (9) the appropriateness of the sample size as determined by power analyses. We note that the absence of scoring methods for creative measurements meant that we were unable to assess the quality of creativity tasks here and instead the implementation of creativity measures is simply detailed below (see Tables 2 and 3). Using the descriptions and scoring (detailed in Appendix E of the Supplemental Material), two authors independently scored each sample from both meta-analyses for all nine items (see Appendix F of the Supplemental Material for scoring of studies utilizing control groups designs, and Appendix G of the Supplemental Material for scoring of studies using pretest–posttest designs). Key findings from the quality assessment are outlined below.

**Table 2:** Summary of studies included in the meta-analysis that utilized control group designs

Authors	Date of Publication	Mindfulness Intervention	Creativity Task	Sample Size	Age Range (mean, *SD*)	Control Group	Outcome impact on creativity (significant vs. not significant)	Effect size (Cohen’s *d*; CI 95%)
Zabelina et al.	2011	10-min intervention	TTCT	81	20.5, 0.08	Historical events podcast	Not significant (*p* = .85)	−0.04
Ren et al.	2011	20-min intervention	Insight problems	48	23.3, 0.04	No treatment	Significant (*p* < .001)	0.11
Ostafin and Kassman	2012	10-min intervention	Insight problems	71	19.3, 0.78	10 min audio	Significant (*p* = .03)	0.53
Ostafin and Kassman	2012	10-min intervention	Noninsight problems	71	19.3, 0.78	10 min audio	Not significant (*p* = .69)	0.19
Colzato et al.	2012	3 × 45-min sessions	AUT	19	42.5, 1.02	Open monitoring	Not significant (*p* = .21)	0.10
Colzato et al.	2012	3 × 45-min sessions	RAT	19	42.5, 1.02	Open monitoring	Significant (*p* < .001)	0.77
Walsh	2013	10-min intervention	Insight problems	40	22.22, 7.63	Sham meditation	Not significant *(p* = .28)	0.81
Baas et al.	2014	8-week intervention	AUT	84	23.77, 3.76	No treatment	Significant (*p* < .001)	0.24
Justo et al.	2014	10-week intervention	TTCT	49	16.45, 0.78	Waiting-list control	Significant (*p* < .001)	1.00
Justo et al.	2014	10-week intervention	AUT	49	16.45, 0.78	Waiting-list control	Significant (*p* < .001)	1.28
Ding et al.	2014	1-week intervention	TTCT	40	21.2, 1.3	Integrative body mind training	Significant (*p* < .001)	0.17
Ding et al.	2015b	1-week intervention	TTCT	84	21.8, 1.54	Integrative body mind training	Significant (*p* < .001)	0.82
Gouda et al.	2016	8-week MBSR	TTCT	29	16.2, 0.51	Waiting-list control	Not significant (*p* = .12)	0.54
Gouda et al.	2016	8-week MBSR	TTCT	29	45.9, 8.52	Waiting-list control	Not significant (*p* = .63)	0.02
Müller et al.	2016	20-min intervention	AUT	39	32.82, 12.6	Concentrative task	Not significant (*p* = .26)	0.29
Poure	2016	5-min intervention	RAT	40	20.07, 0.72	Sham meditation	Not significant (*p* = .71)	0.00
Poure	2016	5-min intervention	AUT	40	20.07, 0.72	Sham meditation	Not significant (*p* = .87)	0.03
Poure	2016	5-min intervention	RWT	40	20.07, 0.72	Sham meditation	Significant (*p* = .02)	0.18
Colzato et al.	2017	40-min intervention	AUT	40	42.5, 1.02	Open monitoring meditation	Not significant (*p* = .59)	0.04
Baas et al.	2020	8.5-min intervention	Group session	114	22.2, 4.08	AWA mediation	Significant (*p* = .02)	0.47

Abbreviations included in Table 2 are as follows: Torrance Test of Creative Thinking (TTCT; Torrance, 1984); Alternative Uses Task (AUT; Guilford, 1967); Remote Associates Task (RAT; Mednick, 1962); Real-World Task (RWT; Poure, 2016).

**Table 3:** Summary of studies included in the meta-analysis that utilized pretest–posttest designs

Authors	Date of Publication	Mindfulness Intervention	Creativity Task	Sample Size	Age Range (mean, *SD*)	Outcome impact on creativity (sig/n.sig)	Effect size (CI 95%)
Justo	2009	Meditation programme	Fluency, flexibility, and originality	60	17.3, 0.18	Significant (*p* = .004)	1.65
Ren et al.	2011	20-min intervention	Insight problems	48	23.3, 0.04	Not significant (*p* = .051)	0.28
Ostafin and Kassman	2012	10-min intervention	Insight problems	71	19.3, 0.78	Significant (*p* = .02)	0.53
Ostafin and Kassman	2012	10-min intervention	Noninsight Problems	71	19.3, 0.78	Nonsignificant (*p* = .93)	0.19
Shapiro et al.	2012	8-week MBSR	Decision-making task	25	32.3, 0.98	Not-significant (*p* = .30)	0.17
Walsh	2013	10-min intervention	Insight problems	40	22.22, 7.63	Not significant (*p* = .21)	0.8
Justo et al.	2014	10-week intervention	TTCT	49	16.45, 0.78	Significant (*p* < .001)	1.92
Baas et al.	2014	8-week intervention	AUT	84	23.77, 3.76	Not significant (*p* = .63)	0.24
Ding et al.	2015a	1-week intervention	TTCT	40	21.2, 1.3	Significant (*p* < .001)	0.81
Ding et al.	2015b	1-week intervention	TTCT	40	21.5, 1.45	Significant (*p* < .001)	0.12
Ding et al.	2014	1-week intervention	TTCT	84	21.8, 1.54	Significant (*p* < .001)	0.82
Gouda et al.	2016	8-week MBSR	TTCT	29	16.2, 0.51	Not significant (*p* = .63)	0.02
Müller et al.	2016	20-min intervention	AUT	39	32.82, 12.26	Significant (*p* = .02)	0.15
Poure	2016	5-min intervention	RAT	40	20.07, 0.72	Not significant (*p* = .71)	0.00
Poure	2016	5-min intervention	AUT	40	20.07, 0.72	Not significant (*p* = .33)	0.03
Poure	2016	5-min intervention	RWT	40	20.07, 0.72	Significant (*p* = .01)	0.18
Berkovich-Ohana et al.	2017	15-min intervention	AUT	48	41.7, 11.43	Significant (*p* < .001)	0.68

Abbreviations included in Table 3 are as follows: Torrance Test of Creative Thinking (TTCT; Torrance, 1984); Alternative Uses Task (AUT; Carroll & Guilford, 1968); Remote Associates Task (RAT; Mednick, 1962); Real-World Task (RWT; Poure, 2016)

With the exception of Baas et al. (2020), studies included in the current meta-analyses have not conducted an adequate power analysis to determine an appropriate sample size. We did not formally conduct power analyses for these studies ourselves, yet inspection of them would suggest that some are underpowered, such that no firm conclusions can be drawn from these individual studies, further justifying the use of the current meta-analytical approach. We also note that many studies fail to measure mindfulness using published questionnaires, raising concerns as to whether the changes in creativity reported are in line with increased mindfulness. It is also unclear whether participants differed in mindfulness traits (e.g., observing, acceptance) and how these individual differences might influence creative performance.

Most studies report a random allocation procedure, although specific information about such procedures is not detailed. For example, simple randomization assigns subjects to each group based on an allocated number (e.g., even = intervention group, odd = control group). In contrast, stratified randomization factors in confounding variables (e.g., prior meditation experience) in order to equate participants across conditions. Without these specific details, we cannot be certain that researchers are accurately performing randomization techniques, which is a common issue across the psychological literature (Kim & Shin, 2014). Furthermore, few studies assess baseline group differences prior to grouping, and although groups appear to be matched based on participant demographics for most studies, there is nevertheless no record of the assessment and grouping procedure. Despite significance testing of baseline differences in RCTs not being required in recent years, De Boer et al. (2015) continue to recommend adjusting for known or anticipated important prognostic variables (e.g., prior meditation experience), as there is evidence that the effect of mindfulness interventions on attentional processes may differ between novice and experienced mindfulness practitioners (Becerra et al., 2017).

### Coding procedure

The moderator variables described previously are viewed as being fundamental to discriminating between differing theoretical accounts of the link between mindfulness and creativity. The procedure that we adopted for coding these moderator variables is presented in Table 4. Mindfulness intervention length was categorized into short (intervention under 20 min), medium (intervention between 20 min but not exceeding 1 week, inclusive), and long (intervention exceeding 1 week). Creative tasks were coded as either convergent thinking tasks or divergent thinking tasks using established criteria for categorizing such tasks in the literature. Finally, in the first meta-analysis only, control group was categorized as active control group, waiting-list control group or no-treatment control group.

**Table 4:** Coding procedure for moderator variables

Moderator Variable	Codes
Control group (first analysis only)	1 = *active* control group 2 = *waiting-list* control group 3 = *no-treatment* control group
Type of creativity task	1 = *convergent thinking task* 2 = *divergent thinking task*
Mindfulness intervention length	1 = *under 20 min* 2 = *over 20 min and less than one week (inclusive)* 3 = *more than 1 week*

### Effect-size calculation

The CMA software was utilized to calculate an effect size, Cohen’s *d*, for each study (Bornstein et al., 2013). In the analysis of studies utilizing control groups, Cohen’s *d* comprised the difference in mean creativity scores between the control and intervention conditions divided by their pooled standard deviation (Freeman et al., 1986). In the analysis of studies using pretest–posttest designs, Cohen’s *d* comprised the difference in mean creativity scores between pre- and postintervention conditions, divided by their pooled standard deviation. In some cases, effect sizes had to be calculated from *t* and *F* values, frequencies, or *p*-values, in which case Wilson’s Effect-Size Calculator was used (Lipsey & Wilson, 2001). If authors noted the *p*-less-than value rather than exact value, then the *p*-less-than value was treated as the exact value (cf. Sio & Ormerod, 2009). Primary effect sizes were calculated for each study; if a study used multiple time points to assess postintervention effects, then the first reported posttreatment time point was included (time points ranged from immediately postintervention to 3 months postintervention). For studies that reported subscales for creativity, an average effect size was calculated.

### Random effects model

The current meta-analyses only used random effects models (see Bornstein et al., 2013). Fixed effects models assume that there is one true effect size that underlies all the studies in the analyses, and that all effect sizes of a given association approximate this effect. This assumption is rarely true in meta-analyses and can therefore lead to inaccurate effect-size estimates due to a high degree of heterogeneity between effect sizes. Heterogeneity is quantified with a statistic that is referred to as *I*^*2*^. Guidelines suggest that *I*^*2*^ values of 0% to 30% indicate low heterogeneity, 31% to 60% indicate medium heterogeneity, and 61% to 100% indicate high heterogeneity (Higgins et al., 2003). To justify the use of random effects models in the current meta-analyses, we provide *I*^*2*^ values for all main analyses. Random effects models are recommended over fixed effects models for most meta-analyses, especially those incorporating RCTs. Given the low to medium heterogeneity present in both samples of the current meta-analyses (see Results section), random effects models were deemed to be necessary.

## Meta-analysis of control group studies

### Overall description of the literature

All studies utilized a mindfulness intervention that ranged from a 5-min session to a 10-week course. These were categorized in the current analysis into short interventions (under 20 min; *n* = 8), medium interventions (over 20 min but under 1 week, inclusive; *n* = 7) and long interventions (more than 1 week; *n* = 5). Interventions were delivered in either group (*n* = 6) or individual (*n* = 14) formats, with all long interventions delivered in group formats. All interventions led participants through exercises based on classic mindfulness instructions proposed by Kabat-Zinn (1990). These exercises included breath focusing, body scans (focusing on each body area, in turn, from head to toe) and mindful stretching. All studies utilized a control group design.

Creativity measures varied between both convergent thinking tasks (*n* = 8) and divergent thinking tasks (*n* = 12). Five studies utilized the AUT as a divergent thinking task. Three studies utilized the RAT or CRAT as a convergent thinking task. Six studies utilized the TTCT as a divergent thinking task, whereby participants are scored on several aspects including creative titles, pictures, expressions and imagery. Four studies used various insight problems that arguably require both convergent and divergent thinking. These insight problems were, however, categorized as convergent thinking tasks in the present analysis, based on Webb et al.’s (2017) proposal that convergent thinking is more important than divergent thinking when tackling problems that are typically solved via insight. One study used a variety of problems that can be classified as divergent thinking tasks. A further study used a Real-World Task (RWT; Poure, 2016), which parallels the AUT conceptually and can therefore be categorized as a divergent thinking task. Finally, the remaining study used a group creativity problem that involved the search for a single solution, which can, therefore, be described as a convergent thinking task.

### Preliminary analyses

**Fig. 1:**
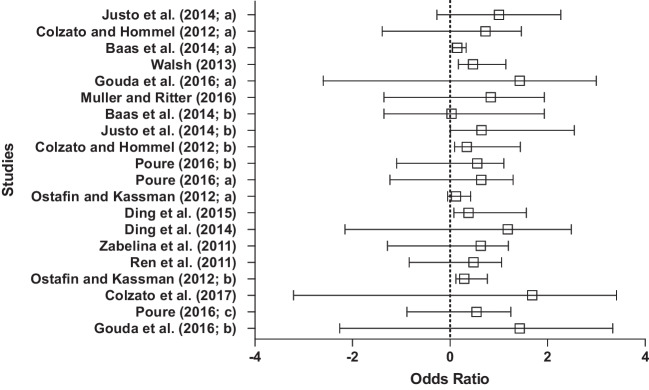
Stem-and-leaf display of the distribution of the effect sizes for studies utilizing control groups

Study characteristics and results were used to create a stem-and-leaf display showing the distribution of the effect sizes (see Fig. 1). Most of the plots fall on the right side of the graph, indicating that in each of the studies the participants’ creative performance was better in the intervention group compared with the control group. Heterogeneity can be quantified in a random effects model by using the *Q*-statistic (to test the assumption of heterogeneity), the *T*^*2*^ value (to examine between-study variance) and the *I*^*2*^ value. A significant *Q* statistic, 34.35, *p* < .001, indicated a heterogeneous distribution of effect sizes, with *T*^*2*^ = 0.016. The *I*^*2*^ value showed that 44.68% of the variance could be attributed to between-study variance. Several variables could be responsible for this, which will be further considered in the discussion. Publication bias was considered low for this sample (see Table 1).

### Results

Twenty studies utilizing control groups were included in the meta-analysis, with a total of 1,036 participants (*Mdn* = 40). Effect size estimates (Cohen’s *d;* see Cohen, 1992) were computed for each independent study, whereby all reported positive effect sizes, ranging from 0.004 to 1.28 (*Mdn =* 0.24; refer to the “CGRD” Supplemental Material for the raw data). Figure 1 presents a stem-and-leaf display showing the distribution of these effect sizes. The unweighted mean of the adjusted effect size estimate was 0.42. The upper and the lower bounds of the 95% confidence interval were 0.29 and 0.54. The confidence interval does not include zero, implying that the estimate of the mean effect size is significantly larger than zero. Using CMA, a random effects model was selected to run meta-regression analyses (Field & Gillett, 2010; see Supplemental Material “MR” for the meta-regression analyses). The mean effect size of the studies utilizing control groups was significant, *d* = 0.42 (0.004 to 1.28; see Fig. 1 for the distribution), *p* < .001, 95% CIs [0.292, 0.544], indicating that mindfulness interventions are moderately successful at improving creative performance scores.

Following recommendation from Bornstein et al. (2013), moderation analyses were completed using subgroup analysis for all categorical moderators (control group, creativity type and intervention length; see Appendix K of the Supplemental Material for random effects data; see Appendix L of the Supplemental Material for moderator analyses).

Moderator analysis on control groups revealed significant mean effect sizes for active control groups, *d* = 0.47, *p* < .001, 95% CIs [0.37, 0.56], for waiting-list control groups, *d* = 1.01, *p* < .001, 95% CIs [0.192, 1.828], and for no-treatment control groups, *d* = 0.24, *p* < .001, 95% CIs [0.15, 0.33]. Subgroup analysis revealed a significant difference between types of control groups, *Q* = 13.93, *df* = 2, *p* < .001. Waiting-list control groups showed a large effect size (*d* = 1.01), active control groups showed a small to medium effect size (*d* = 0.47), and no-treatment control groups showed a small effect size (*d* = 0.24).

Moderator analysis revealed significant mean effect sizes for convergent thinking tasks, *d* = 0.47, *p* < .01, 95% CIs [0.32, 0.61], and for divergent thinking tasks, *d* = 0.25, *p* =.001, 95% CIs [0.16, 0.34]. Subgroup analysis revealed a significant difference between the type of creative task, *Q* = 5.87, *df* = 1, *p* < .001, suggesting mindfulness is more effective at improving problem-solving success for convergent thinking tasks, with a small to medium effect size (*d* = 0.47), compared with divergent thinking tasks, with a small effect size (*d* = 0.25).

The final moderator analysis focused on intervention length and revealed significant mean effect sizes for intervention length as follows: short intervention, *d* = 0.44, *p* < .001, 95% CIs [0.30, 0.59], medium intervention, *d* = 0.55, *p* < .001, 95% CIs [0.16, 0.95], and long intervention, *d* = 0.25, *p* < .001, 95% CIs [0.16, 0.34]. The overall subgroup analysis demonstrated a significant difference between the intervention types, *Q* = 6.20, *df* = 2, *p* < .001. By observing effect sizes, all intervention lengths were effective at improving creativity scores, with medium length (20 min to 1 week) offering a medium effect size (*d* = 0.55), followed by short interventions offering a small to medium effect size (*d* = 0.44), and finally long interventions, offering a small effect size (*d* = 0.25).

## Meta-analysis of pretest–posttest studies

### Overall description of the literature

All studies utilized a mindfulness intervention, which ranged from a 5-min session to a 10-week course. These were categorized in the current analysis into short interventions (under 20 min; *n* = 6), medium interventions (over 20 min but under 1 week, inclusive; *n* = 6) and long interventions (more than 1 week; *n* = 5). Interventions were delivered in either group (*n* = 4) or individual (*n* = 13) formats. All interventions led participants through exercises based on classic mindfulness instructions proposed by Kabat-Zinn (1990). This included breath focusing, body scans (focusing on each body area, in turn, from head to toe), and mindful stretching. Creativity measures varied between both convergent thinking tasks (*n* = 5) and divergent thinking tasks (*n* = 12). Four studies utilized the AUT, one utilized the RAT, five utilized the TTCT, three used problems that are typically solved via insight, one study used noninsight problems, one study used an RWT, one used a decision-making task, and the final study used a creative originality task which shares similarities to the AUT.

### Preliminary analyses

**Fig. 2:**
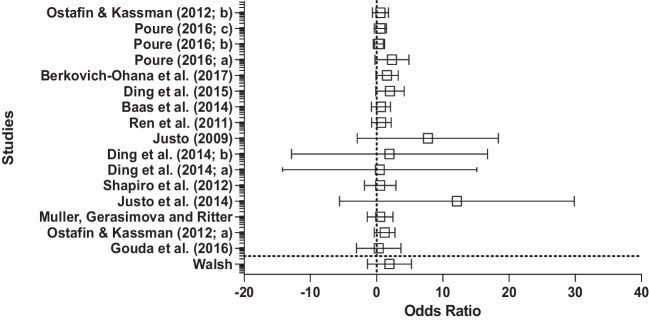
Stem-and-leaf display of the distribution of the effect sizes for studies utilizing a pretest–posttest design

Study characteristics and results were used to create a stem-and-leaf display showing the distribution of the effect sizes (see Fig. 2), which indicates that in each of the trials, the participants’ creative performance was better postintervention compared with pre-intervention. A nonsignificant *Q* statistic (*Q =* 14.94, *p* = .53) indicated a nonheterogeneous distribution of effect sizes, *T*^*2*^ = 0.62. The *I*^*2*^ value showed that there is no observed heterogeneity in this sample. Standard error and standard difference in means were used to create a funnel plot to assess the potential role of publication bias (Harbord et al., 2006; see Appendix D of the Supplemental Material). Observational analysis reveals asymmetry in the funnel plot, indicative of publication bias. Trim and fill analyses, a nonparametric data augmentation technique, was therefore used to adjust for identified publication bias in the sample by yielding adjusted effect sizes for this dataset (Duval & Tweedie, 2000).

### Results

Seventeen studies utilizing pretest–posttest designs were included in the meta-analysis, with a total of 848 participants (*Mdn* = 25), and all 17 reported positive effect sizes ranging from 0.004 to 1.92 (*Mdn* = 0.24; refer to the “PPRD” Supplemental Material for raw data). Figure 2 presents a stem-and-leaf display showing the distribution of these effect sizes. The unweighted mean of the adjusted effect size estimate was 0.59, with a standard deviation of 0.11. The upper and the lower bounds of the 95% confidence interval were 0.38 and 0.81. The results from the meta-regression analyses using a random effects model, *p* < .001, 95% CIs [0.38, 0.81], suggest that mindfulness interventions were highly successful at improving creative performance scores (see Appendix J of the Supplemental Material for random effects data; see Appendix M of the Supplemental Material for moderator analyses; see Supplemental Material “MR” for meta-regression analyses), with the mean effect size of studies utilizing pretest–posttest designs being significant, *d* = 0.59 (0.004 to 1.92; see Fig. 2 for the distribution).

The first moderator analysis focused on creativity type and revealed significant mean effect sizes for convergent thinking tasks, *d* = 0.80, *p* < .001, 95% CIs [0.35, 1.2], and divergent thinking tasks, *d* = 0.52, *p* < .001, 95% CIs [*0*.24, 0.80]. Subgroup analysis revealed a nonsignificant difference between the type of creativity, *Q* = 1.04, *df* = 1, *p* = .53. Mindfulness may be more effective at improving performance on convergent thinking tasks, with a medium to large effect size (*d* = 0.80), compared with divergent thinking tasks, with a medium effect size (*d* = 0.52); however, this difference was not significant.

Moderator analysis for intervention length revealed mean effect sizes for intervention length as follows: short intervention, *d* = 0.45, *p* = .002, 95% CIs [0.17, 0.73], medium intervention, *d* = 0.73, *p* < .001, 95% CIs [0.33, 1.14], and long intervention, *d* = 1.06, *p* = .002, 95% CIs [0.23, 1.87]. The overall sub-group analysis demonstrated no significant difference between the intervention lengths, *Q* = 2.64, *df* = 2,* p* = .27. Therefore, it can be concluded that intervention length did not impact intervention success on creative performance. However, longer interventions led to a larger effect size (*d* = 1.06), relative to medium interventions (medium to large effect size: *d* = 0.73) and short interventions (small to medium effect size: *d* = 0.45).

## Discussion

The contradictory findings in the literature relating to the influence of mindfulness on creativity warranted further investigation, thereby necessitating the current meta-analyses. Commonly held criticisms concerning existing studies examining the effect of mindfulness on creativity have noted the lack of control groups (e.g., Baer, 2003; Bishop, 2002; Lebuda et al., 2016), hence the present analyses focused on the use of control groups as a moderator along with intervention length and the type of creativity task in terms of the predominant distinction in the literature between divergent thinking tasks and convergent thinking tasks. It was deemed to be especially important to differentiate between different study designs with respect to the control-group moderator to tease out the influence of control groups in the mindfulness literature relative to the use of pretest–posttest designs.

Consistent with previous findings (Capurso et al., 2014; Ding et al., 2014; Henriksen et al., 2020), we provide support for the overall effectiveness of mindfulness interventions in enhancing creative performance. Here, mindfulness significantly improved creativity with a small to medium effect size for studies with a control group design (*d* = 0.42) and with a medium effect size for pretest–posttest studies (*d* = 0.59). The differences in the observed effect sizes further justifies the use of two separate meta-analyses here and emphasizes the importance of study design in mindfulness intervention studies. Control group designs are often favoured to pretest–posttest designs, specifically in relation to RCTs, due to the ability to control for the influence of demand characteristics and placebo effects (Baer, 2003). For this reason, our expectations were for control group studies to report larger effect sizes due to the superior methodological design. Unexpectedly, pretest–posttest design studies displayed a larger effect size compared with control group design studies. We therefore outline possible explanations for this result below.

First, practice effects are a common issue highlighted in pretest–posttest designs, and it is possible that observed improvements are a result of task repetition (Dimitrov & Rumrill, 2003). Several studies (Ostafin & Kassman, 2012; Poure, 2016; Walsh, 2013) also failed to provide a measure of state or trait mindfulness before or following the intervention, therefore it is unclear whether mindfulness characteristics increased in line with creative performance, as indicated by Baas et al. (2014), or whether there was no correlation between the two, as suggested by Zedelius and Schooler (2015). Furthermore, specific studies (e.g., Justo et al., 2014) reported a very large effect size of 1.92, and it is possible that this study alone may have influenced the overall effect size of the pretest–posttest analysis. It is important to note that although adjustments were made to the pretest–posttest data using Duval and Tweedie’s (2000) trim and fill method, publication bias was still detected both in observational analysis of the funnel plot and via Begg and Mazumdar’s Rank Correlation Test, suggesting the possibility of missing studies, which might have altered the overall interpretation of the current review, and conclusions should therefore be treated cautiously due to the low quality and volume of studies. Finally, the implication of the larger effect size of pretest–posttest studies relative to that for the active control groups might be that the latter studies were better at identifying benefits of mindfulness while excluding other aspects of the intervention, therefore in that sense, active control groups were more effective than pretest–posttest designs, in line with the literature (e.g., Baer, 2003).

### Active versus no-treatment versus waiting-list control groups

To restate, a key methodological limitation of existing mindfulness research is the type of control group that is selected in an effort to ensure that observed results are due to a mindfulness manipulation. Several researchers have raised concerns, mainly towards the use of waiting-list control groups (e.g., Park et al., 2013), although criticisms also extend to poorly chosen active control groups (MacCoon et al., 2012), and to no-treatment control groups (Farias & Wikholm, 2016). It was, therefore, important to analyze the types of control groups utilized in the current review of studies, and to assess how each type of control group might influence findings.

Here, we found a significant difference between the types of control groups used to assess creative performance, validating the approach to the current meta-analyses. Bishop (2002) and Baer (2003) highlighted criticisms in the mindfulness literature relating to the lack of active control groups. Baer (2003) noted that although treatment-as-usual controls and no-treatment controls account for change due to time, an active control is needed to account for the influence of demand characteristics and placebo effects, and specifically allows for the interrogation of the benefits of mindfulness practices as opposed to the benefits of relaxation, which may have a confounding effect. In support of these concerns, the current review reports a medium effect size using active control groups (*d* = 0.47) and a small effect size using no-treatment control groups (*d* = 0.24).

The active control groups (*n* = 14) utilized in the analyses ranged from a podcast on an irrelevant topic (Zabelina et al., 2011), to an integrative body-and-mind training session (Ding et al., 2015a). This variance in active control tasks has the potential to increase confounding variables, for example, mind wandering, which plays a role in creative performance and varies dependent on the demand of a task (e.g., a podcast could be viewed as being a low-demand task whereas body-and mind-training could be viewed as being a high-demand task). Task demand seems to be important for mind wandering and creative performance (e.g., higher task difficulty can lead to higher mind wandering and impasse), and therefore may influence supposed effects of creativity on mindfulness.

The current review found waiting-list control groups to yield the largest effect size (*d* = 1.01). There are some ethical advantages of employing waiting-list designs in that they allow for participants who are seeking help, perhaps for clinical interventions, to access the same level of care as the intervention group, whilst eliminating the use of a “sham” or “placebo” intervention. There are also some theoretical arguments for favouring the use of waiting-list control groups (e.g., because they provide an untreated comparison condition using participants who closely resemble those in an intervention group), but there have been observations (e.g., Cunningham et al., 2013) that waiting-list control groups can overestimate, or artificially inflate, intervention effects, in that waiting-list control groups may not control for extraneous variables that systematically relate to the desired variable, in our case, mindfulness.

Cunningham et al. (2013) conducted an exploratory randomized trial to test the proposition that waiting-list control groups may artificially inflate intervention effect estimates as earlier suggested (cf. Hart & Bagiella, 2012; Hart et al., 2008), and gave further weight to the need for caution in employing waiting-list designs, providing evidence of waiting-list conditions overestimating treatment effects. Cunningham et al. (2013) highlighted several explanations for this overestimation effect, leaning towards the likelihood that waiting-list controls interrupt readiness for, or activities towards, changing behaviour. Participants placed on a waiting list for active treatment may be less likely to engage in beneficial self-help whilst they are waiting than a group who were not waiting for treatment. Because the waiting list control group do less self-help, then there seems to be a larger difference between them and the treatment group, hence a bigger effect size than observed in other control group designs.

Although the waiting-list control groups included in the current sample were allocated randomly, and were assumed to be comparable to experimental groups, most of the studies did not tease out between-group differences in their samples (e.g., Ostafin & Kassman, 2012), and it is possible that group differences in creative performance and/or mindfulness traits existed prior to the intervention, influencing findings. We suspect that some waiting-list control groups employed here could have artificially inflated the effect sizes of the mindfulness intervention, for the reasons outlined above. In sum, waiting-list control groups seem less desirable than other controls for multiple reasons, but mainly due to their inability to control for extraneous variables, and we therefore support the view that waiting-list control groups should only be employed when it would be unethical to deny participants access to treatment (Ovosi et al., 2017).

Overall, researchers do seem to be addressing concerns surrounding active control groups (Baer, 2003; Bishop, 2002; MacCoon et al., 2012); however, there still exists a limitation in that active-control groups are too varied and broad, and a consensus concerning a clear definition of active-control groups is required to ensure consistency across studies. We support MacCoon et al.’s (2012) definition and recommend that future research should utilize active control tasks that match mindfulness interventions in nonspecific factors (e.g., duration) and not include mindfulness, to ensure that group differences can be attributed to the absence or presence of the experimental condition, ensuring consistency across studies.

### Convergent versus divergent thinking tasks

Mindfulness has been linked with processes that might be beneficial for creativity, including attentional control and executive functioning (Anderson et al., 2007; Tang et al., 2012). Such claimed benefits arising from mindfulness are, however, contested and might also depend on the convergent or divergent nature of the creativity task at hand (for a review, see Lebuda et al., 2016). From a theoretical standpoint, therefore, it was important in the present meta-analyses to differentiate between convergent and divergent thinking tasks.

In line with previous research, mindfulness significantly improved tasks assumed to measure creativity, with a small to medium effect size being observed for studies with a control group design, and a medium effect size for studies utilizing a pretest–posttest design. The type of creativity task did emerge as a significant moderator for studies utilizing control groups. More specifically, mindfulness was more effective at improving creative performance on convergent thinking tasks (*d* = 0.47) compared with creative performance on divergent thinking tasks (*d* = 0.25). For studies utilizing pretest–posttest designs, there was no significant moderator effect of creativity task, suggesting mindfulness improves creativity task performance regardless of whether creative performance is assessed through the deployment of convergent or divergent thinking tasks. However, despite no significant difference being reported, the pattern of effect sizes suggested that mindfulness is, again, more effective at improving performance on convergent thinking tasks (*d* = 0.80) than on divergent thinking tasks (*d* = 0.52).

In comparison with our other moderator variables, methodological differences in study design do not seem to change the beneficial effects of mindfulness on overall creative performance to a large degree. Despite only control-group studies eliciting a significant difference between convergent and divergent creativity tasks, there are still clear patterns emerging overall to suggest that mindfulness enhances performance more on the former than the latter. This finding points toward the idea that the effects of mindfulness on convergent versus divergent thinking tasks may rely on different functional and neural mechanisms.

To reiterate, divergent thinking tasks are commonly viewed as drawing upon two core processes—that is, idea generation and idea evaluation (Runco, 2012; Sowden et al., 2015). At a more detailed level, divergent thinking tasks appear to involve an interplay between Type 1 associative processes that are dominant during idea generation and Type 2 analytic processes that are dependent upon attention and WM, which are prevalent during idea evaluation. These processes, moreover, are commonly linked, respectively, to the functioning of the DMN and ECN (Beaty et al., 2015), whereby cognitive switching between networks is particularly beneficial for divergent thinking. Mindfulness is also associated with reduced activation of the DMN as well as with enhanced executive and attentional control that is associated with activation of the ECN (Taren et al., 2017), supporting the notion that synchrony between DMN and ECN is required for divergent thinking.

This involvement of attentional and executive processes, leading to better idea evaluation, enables us to make sense of the benefits that can arise from mindfulness interventions on divergent thinking. The current review supports the possibility that mindfulness enhances performance on divergent thinking tasks such as the AUT by increasing the efficiency of brain networks such as the ECN, thereby enhancing executive aspects of attentional processing that are required to do well on such tasks. In terms of Type 1 and Type 2 processing, our results therefore indicate that Type 2 processing is more relevant for the idea evaluation component that is important for successful performance on divergent thinking tasks. Individuals need to draw upon WM and cognitive decoupling, as well as mental stimulation to appraise the quality of divergent outcomes.

As in the case of processing on divergent thinking tasks, attentional control may also be a key requirement for effective performance on convergent thinking tasks, especially in terms of the involvement of WM capacity, which relates to the set of attentional processes that enable individuals to maintain goals, prioritize relevant information and avoid distractions (Oberauer, 2019). According to the business-as-usual view of insight problem-solving with convergent thinking tasks (e.g., Ball & Stevens, 2009; Gilhooly et al., 2015), individuals draw upon WM resources to plan and execute search strategies to reach a solution as well as to evaluate solution success (e.g., MacGregor et al., 2001; Marsh et al., 2021; Ormerod et al., 2002, 2013; Payne & Duggan, 2011). Attentional control and WM capacity are also enhanced by mindfulness practice (Taren et al., 2017), by activating the same brain regions and allowing them to work more efficiently (Bailey et al., 2019). It therefore seems plausible that attentional and WM processes play an important role in understanding how mindfulness impacts creative outcomes with convergent thinking tasks.

On the other hand, the special-process-view of insight problem-solving with convergent thinking tasks (e.g., Bowden et al., 2005; Gilhooly et al., 2015) suggests that solutions are achieved through an open-minded approach that is primarily driven by nonconscious restructuring and associative processes. Based on this view, mind-wandering frequency, which improves one’s ability to be more open-minded and elicit nonconscious processes, has been shown to be correlated with higher creative performance (cf. Yamaoka & Yukawa, 2020). More specifically, mind wandering has been claimed to be important on convergent thinking tasks (Baird et al., 2012; Zedelius & Schooler, 2015), as is evidenced by studies investigating incubation effects, which demonstrate that mind wandering during incubation enhances the attainment of convergent task solutions (Baird et al., 2012; Leszczynski et al., 2017). In applying the proposed mechanism of mind wandering in conjunction with the special-process-view, then the prediction would be that performance on convergent thinking tasks should decrease following a mindfulness intervention because the attentional control that arises is counterproductive for nonconscious restructuring and associative processing.

However, our findings support the notion that mindfulness improves creative performance on convergent thinking tasks. Critically, mindfulness has been found to reduce mind-wandering thoughts, encouraging heightened present-moment awareness and attention (Xu et al., 2017). We also note here an alternative explanation of our findings, which is that the act of practicing mindfulness might improve the cognitive control required to switch flexibly between defocused attention (in alignment with the special-process view) and focused attention (in alignment with the business-as-usual view). We suggest that future research would do well to examine this proposal that enhanced cognitive flexibility might have a role to play in understanding the link between mindfulness and creativity on convergent thinking tasks, and potentially on divergent thinking tasks too.

Notwithstanding our interpretation of the evidence as seemingly supporting a business-as-usual account of creative processing on convergent thinking tasks, we also recognize that within any one study using such tasks there will be numerous trials, each involving a different problem (e.g., a RAT, CRAT, or rebus item), with some problems perhaps being solved in a business-as-usual way but others being solved in a special-process way. It would, therefore, only be aggregate data, which collapses across individual problems, that would reveal an overall benefit of mindfulness because of the enhancement effects arising solely on the problems solved in a business-as-usual manner. Under this account, it would have to be assumed that mindfulness interventions have little negative impact when problems are solved via a special-process mechanism, which is entirely possible. We contend that future research would benefit from examining more carefully the links between mindfulness practice and task success at the item level, where neuroimaging evidence and phenomenological self-reports could be used to determine whether any particular problem has been solved either in a more step-by-step and analytic way (in line with the business-as-usual account) or in a more holistic and intuitive way (in line with the special-process account).

A further important point to consider in interpreting data relating to a potential link between mindfulness and creativity is that mindfulness practice might have a beneficial effect on aspects of cognition other than attention and WM, which might likewise indirectly translate into improvements in creative performance. For example, many researchers testify to the impact that mood has on creativity and argue that creative tasks are mood sensitive (e.g., Amabile et al., 2005), reporting a significant, positive relationship between elevated mood states and creativity (e.g., Davis, 2009). There is likewise an extant literature that supports a positive association between mindfulness and enhanced mood (e.g., Garland & Howard, 2013; Keng et al., 2011). Therefore, it is not unreasonable to suggest that the benefits that arise from mindfulness on creative performance could be mediated by positive mood.

In a similar vein, the construct of mindfulness has been claimed to promote single-minded goal maintenance, which has been theorized to relate to the goal maintenance component of the cognitive control element of working memory (Gazzaley et al., 2005). Individuals can also improve their ability to encode information through mindfulness practice (Lueke & Lueke, 2019). In sum, we accept that the benefits of positive mood, goal maintenance, and problem encoding on creative cognition could potentially account for the beneficial effect of mindfulness on creativity reported here, and further research is clearly needed to investigate this possibility.

### Outcomes of short, medium and long mindfulness interventions

For pretest–posttest studies, there was no significant difference between intervention lengths, suggesting intervention length did not affect the success of an intervention on creative performance. Here, long interventions were most effective at improving creative performance with a large effect size (*d* = 1.06), followed by medium interventions (*d* = 0.73) and short interventions (*d* = 0.45). These findings do not run counter to the notion that longer, more intensive mindfulness interventions may be the most effective (Josefsson et al., 2011).

For studies employing a control-group design, however, intervention length did emerge as a significant moderator. Here, medium interventions were most effective at improving creative performance (*d* = 0.55), followed by short interventions (*d* = 0.44) and then long interventions (*d* = 0.25). Despite all three successfully improving creativity, these findings run counter to previous research, which has indicated that longer, more intensive interventions are the most effective (Josefsson et al., 2011). It could be argued that interventions classified as short in the present review (i.e., under 20 min), might not be long enough to elicit any change. Other researchers, such as Jedrczak et al. (1986), have categorized short interventions as less than 1 week. It is possible that our short category did not include interventions that other researchers would have termed short. However, our categorization methods are in line with Mrazek et al. (2012), who suggested that mindfulness interventions effectively stimulate attentional processes, regardless of intervention length, and our observation that all intervention lengths facilitate creative performance likewise supports the views of Mrazek et al. (2012). In addition, our finding that long interventions have the lowest benefit for creative performance is in line with Jedrczak et al. (1986), who found shorter interventions (i.e., less than 1 week) to be more effective than long interventions at improving performance in a range of cognitive tasks.

Furthermore, there was considerable variation in the *intensity* of the interventions studied in Jedrczak et al. (1986), but only intervention length was measured in the current analysis. There is evidence that mindfulness intensity affects outcomes such as attention and stress (Khoury et al., 2015), although others have failed to find such effects (e.g., Carmody & Baer, 2009). Given that increased mindfulness leads to improved attention (Norris et al., 2018), and that improved attention can improve creative processes, it is therefore possible that intervention intensity may have mediated current findings and may explain differences in effectiveness of intervention lengths across studies. Furthermore, it is important to consider the difficulties in the measurement of intensity and adherence, and these factors should be accounted for in future studies.

We contend that the paucity of research examining mindfulness intervention length makes it difficult to determine appropriate groupings. Despite our categorization approach disbursing all 20 studies equitably into short (*n* = 8), medium (*n* = 6) and long (*n* = 6) interventions, we concede that if different categories were selected, findings could vary. We emphasize the need for some form of consensus between researchers regarding the appropriate categorization of mindfulness interventions to ensure consistency across future studies.

### Underpinning processes of the mindfulness–creativity link

There is consistent evidence that there are common cognitive processes that are important for both mindfulness and creativity. However, there has been a lack of consensus on how the two concepts interrelate. Although some evidence indicates that mindfulness is effective in enhancing creative performance, such evidence has been complicated by the existence of research that either contradicts or fails to support a mindfulness–creativity link (see Henriksen et al., 2020). Based on the findings from the current meta-analytical review, however, we conclude that mindfulness influences processes that are important for creative processing on both divergent and convergent thinking tasks.

The neuroscientific research on divergent thinking points towards an interplay between associative processing, linked to the DMN, and attentional processing involved in WM and executive functions, linked to the ECN (Beaty et al., 2020). We propose that mindfulness improves performance on divergent thinking tasks by activating the ECN to enhance the executive aspects of processing (e.g., attention and WM) that are required for evaluative activity in tasks such as the AUT and TTCT, leading to improved task performance.

In relation to convergent thinking tasks, the current meta-analytical review provides support for the business-us-usual account of performance on such tasks, in that attentional control and WM appear to be essential for successful solutions to convergent thinking problems. It is likely that WM capacity can be enhanced by mindfulness practice by activating the same brain regions (Bailey et al., 2019). Therefore, our findings suggest that mindfulness practice enhances attentional processes, which can increase WM capacity, thereby aiding creative problem-solving with convergent thinking tasks.

Hommel (2012) argues that processing on convergent and divergent thinking tasks calls for different cognitive control states, which is a proposal that is in line with our results when considering the larger effect size seen for mindfulness interventions with convergent compared with divergent thinking tasks. Colzato et al. (2012) have provided empirical evidence to support this proposal, by investigating the effects of focused attention and open monitoring meditation, techniques commonly utilized in mindfulness practices, on creative performance. Colzato et al. (2012) reported that focused attention techniques led to improved performance on convergent thinking tasks, indicated by higher scores in the RAT, whereas open-monitoring meditation was more beneficial for divergent thinking tasks, measured using the AUT. Focused attention techniques are typically introduced earlier on in long-term mindfulness programmes and are mostly adopted during short-term practice. This is because a good degree of attentional focus is required to build toward more advanced practices, including open-monitoring techniques, which tend to appear later on in long-term mindfulness programmes and are excluded from shorter interventions. It is, therefore, likely that many of the mindfulness interventions employed in the studies that were included in the current review were more oriented toward focused-attention techniques, thereby leading to the observed higher effect size for convergent thinking tasks compared with divergent thinking tasks.

We also note that the aforementioned proposal is only applicable to experienced meditators, and it is unclear how it might translate to naïve meditators. Researchers suggest that naïve meditators would be more susceptible to interference when performing divergent thinking tasks like the AUT, yet less likely to be affected when tackling convergent thinking tasks (Capurso et al., 2014). If the samples that were included in the current review involved beginners or meditation-naïve participants, then such sampling factors might also account for the larger effect size seen in convergent tasks compared with divergent tasks. These observations remain speculative as researchers typically do not record participants’ level of meditation experience.

A further point to note is that more top-down control would seem be required for performing effectively with convergent thinking tasks than divergent thinking tasks (Fischer & Hommel, 2012). Individual differences in WM capacity also influence top-down processing, as shown by Sobel et al. (2007), who reported that creative searches that relied primarily on top-down mechanisms showed an advantage for participants with higher WM capacity compared with participants with lower WM capacity. It is therefore possible that mindfulness is enhancing WM in individuals by effectively stimulating brain regions associated with WM such as the prefrontal cortex (e.g., Bailey et al., 2019; Mrazek et al., 2013). Participants are therefore advantaged more on convergent thinking tasks that rely primarily on top-down mechanisms than on divergent thinking tasks (Fischer & Hommel, 2012), hence participants undergoing mindfulness practice outperform control participants at creative thinking tasks, and the difference is greater for convergent thinking tasks compared with divergent thinking tasks.

Another explanation worth considering, for the larger effect size seen for convergent thinking tasks compared with divergent thinking tasks, is that the convergent thinking tasks (e.g., RATs and CRATs) are somewhat hybrid in nature, requiring considerable divergent thinking before processing converges on a single, correct solution. Thus, performance on convergent thinking tasks might benefit due to the mindfulness intervention exerting a positive effect on both divergent and convergent thinking processes, as seen from the business-as-usual perspective (Ball & Stevens, 2009). In contrast, the divergent thinking tasks used in some studies (e.g., AUT) are arguably purer measures of only divergent thinking with less convergent thinking required. Although, some convergent processes might be required (e.g., checking that a response is original/unusual on the AUT) this may be less WM intensive, because there is usually no necessity to arrive at the single correct solution, unlike with a convergent thinking task. Thus, although both categories of task might benefit from the impact of mindfulness on the divergent stage of the process, the “convergent” thinking tasks might gain additional benefit from the effect of mindfulness on the convergent stage of the process, due to the beneficial effects on WM.

In sum, our findings seem to support Guilford’s (1967) suggestion that convergent and divergent thinking represent different components of human creativity, and it is likely that there are different underlying cognitive processes involved in these two components. We propose several possible explanations for this, including the influence of top-down processing, the type of mindfulness practice being employed, and the level of meditation experience of the sample. We recommend that further research considers these factors to understand fully the influence of mindfulness on convergent and divergent thinking tasks (Bailey et al., 2019; Mrazek et al., 2013).

Overall, given the dissimilarities between studies included in the current meta-analyses in terms of how mindfulness was operationalized and how creativity was assessed (see Tables 2 and 3 for study details), it is difficult to comment on the reasons for inconsistencies in findings. Here, we have offered some observations and theoretical suggestions, but is seems clear that more conceptual clarity is needed.

## Review of the meta-analytic procedure

As previously discussed, mindfulness is often referred to using a variety of different terms, including “meditation,” “relaxation,” “focused-attention training,” and “open-monitoring training.” This makes it difficult to search and locate papers that are incorporating mindfulness interventions but terming it differently. We recommend that future research should remain vigilant about this variety of terminology in the literature, and we also encourage researchers to work toward developing a consistent and generic concept of mindfulness.

### Limitations of included studies

Several studies in the current sample provided participants with the same problem postintervention, if participants had previously failed to solve the problem preintervention (Colzato et al., 2012; Ren et al., 2011). Not only does this increase practice effects (Calamia et al., 2012) but it also incorporates an accidental incubation period, since a participant’s attention is diverted elsewhere before they are asked to solve the same problem again. Given the compelling evidence that such incubation periods can enhance creativity (Sio & Ormerod, 2009), it is possible that the inadvertent introduction of incubation time in some of the studies included in our meta-analyses may have influenced creative performance as a moderator variable, such that our findings should be interpreted with caution.

Based on Webb et al.’s (2017) recommendation, categorizing tasks into either convergent thinking tasks or divergent thinking tasks is typically based on the processes that are deemed to be most important for tackling them. For example, the AUT is a typical index of divergent thinking that requires combining different types of information in novel and flexible ways (Guilford, 1967). Importantly, some tasks require a combination of both convergent and divergent thinking to reach a solution. Problems that are often solved via insight (for a review see Webb et al., 2017) require the ability to generate diverse ideas (divergent thinking), but more importantly, require the ability to identify a successful solution (convergent thinking). Since convergent thinking is more important here, then such problems would be categorized as convergent tasks, despite requiring some divergent thinking. This overlap in some tasks makes it difficult to categorize tasks with certainty and may account for some variation in findings.

The current meta-analyses are the first to examine the effects of mindfulness interventions on convergent and divergent thinking tasks. To date, only one other review has focused on mindfulness and creativity (Lebuda et al., 2016) and this was limited by a focus on creativity as one holistic construct rather than as involving separable processes that underpin convergent versus divergent thinking tasks. The current review addresses important gaps in the literature relating to the impact of mindfulness on creativity and the role of moderating factors that include the type of creativity task, the length of the mindfulness intervention and the use of control groups. We contend that our findings will benefit future research investigating the mechanisms underpinning mindfulness effects by having helped to clarify some of the main causal dependencies that are likely to exist between manipulated factors.

We acknowledge that the current review is vulnerable to limitations associated with all meta-analyses. Although published guidance was followed (Bornstein et al., 2013; Rothstein et al., 2006), as were previous examples of related meta-analyses in the literature (Lebuda et al., 2016; Sio & Ormerod, 2009), it is still possible that a study has been missed. Nevertheless, publication bias was adjusted where necessary using the trim and fill technique (Duval & Tweedie, 2000) and confidence intervals were adjusted with an estimate of missing trials to account for this. The greatest limitation of the current review is the overall lack of good quality studies focusing on mindfulness interventions with creative outcomes, in turn, leading to a small number of studies in each moderator analysis. The paucity of studies meeting our inclusion criteria therefore limits our ability to draw firm conclusions about mindfulness and creativity.

Importantly, there is also some critique surrounding the use of divergent and convergent thinking tasks of the kind that have been utilized in the current review. For example, Zeng et al. (2011) commented that real-world creativity requires a type of creative processing that is rarely studied in laboratory settings. This raises the question of how researchers can reliably and validly measure an individual’s real-life creative potential. Zeng et al. (2011) confirmed that most researchers resort to psychometric approaches to study convergent thinking (e.g., using the RAT) and divergent thinking (e.g., using the AUT), even though these methods have been criticized (Plucker, 1999). This is because no creative thinking task can independently represent an adequate assessment of individuals’ real-world creativity (Beaty et al., 2015). Real-world creativity cannot occur without the contribution of creative processes, like convergent and divergent thinking (Giancola et al., 2022). Arguably, these tasks on their own do not to represent real-world creativity (Cropley, 2006). Despite such criticisms, divergent and convergent thinking tasks are useful when examining creativity at a more detailed level and are therefore continually utilized in research.

### Quality assessment

All study samples were screened by two individual researchers during the quality assessment procedure, which revealed two key conclusions. First, all studies but one failed to conduct a power analysis to determine sample size and, as a result, some studies appear to be underpowered. It is therefore difficult to draw firm conclusions from the individual studies included in the samples. Second, only eight out of 37 studies included a trait/state mindfulness measure. Thus, it is unclear whether changes in creativity are in line with changes in mindfulness, or instead are due to other variables (e.g., practice effects or individual differences). As discussed in our introduction, self-reported trait mindfulness and experimentally induced mindfulness also elicit different outcomes (Bravo et al., 2018), emphasizing the importance of utilizing an appropriate measure of mindfulness.

### Recommendations for future research

The current review provides support for the use of shorter mindfulness interventions to stimulate improvements in people’s performance with both convergent and divergent thinking tasks. Interventions ranging from 20 min to 1 week were most effective at successfully enhancing creative performance, in line with previous research (Jedrczak et al., 1986; Larson et al., 2013; Mrazek et al., 2012). Importantly, these findings relate to studies in which posttest measurements are taken immediately following the intervention, and results do not account for long-term effects of mindfulness on creativity. Indeed, future research should consider shorter interventions to investigate the effects of mindfulness, thereby allowing for shorter time periods between pretest and posttest measures. This may positively influence sample sizes for intervention-based studies and further understanding of medium length mindfulness interventions. However, studies should also attempt to measure effects weeks, or months, following the intervention, to understand any longevity in the beneficial outcomes of mindfulness practice.

Study designs should also be carefully considered in future research. We note the importance of selecting an appropriate control group due to the inherent limitations of waiting-list control groups given the possibility for these to inflate overall effect estimates, leading to distortion of the true effect for the intervention. Waiting-list control groups seem to be poor for multiple reasons, and we therefore support the view that waiting-list control groups are reserved for circumstances where it would be unethical to deny participants access to treatment (Ovosi et al., 2017). Moreover, we contend that well-formulated, active control groups should be employed as a gold standard based on the goals of the study and its setting. Ideally, it would be valuable if the research community could reach a consensus on suitable active controls for mindfulness interventions, thereby reducing variation across the literature.

As previously mentioned, one main limitation of the control group sample in our meta-analyses is the wide variety of active control groups utilized in the studies reviewed. These ranged from podcasts to an alternative meditation technique. Although these controls do account for the passage of time and for placebo effects (Baer, 2003), they may also influence participant traits such as dispositional mindfulness, which researchers failed to measure in any of the included studies, but which could influence creative performance. Variation in active control groups may have influenced overall effect sizes and may account for waiting-list controls eliciting a larger effect size in the current analysis. We recommend that future studies should continue to utilize control group designs, carefully selecting an appropriate active control, depending on goals and settings, to ensure an appropriate comparison group to test for the effectiveness of mindfulness interventions.

Because of the scarcity of research that adopts a multicomponent approach to mindfulness, our review took a unitary perspective on mindfulness, assuming that it is one, singular construct. This has allowed for advancements in terms of understanding the theoretical underpinnings of the mindfulness–creativity link and this approach also reflects a clear consensus in the literature up until this point. However, we are aware that views of the mindfulness construct are shifting more towards a multicomponent perspective, hence, future research should attempt to adopt this view to deepen understanding of the subfacets of mindfulness.

The way in which researchers define creativity varies considerably. Here, an effort was made to ensure that our definition was broad and inclusive, in line with up-to-date conceptualizations of creativity (e.g., Simonton, 2017, 2018; see also Plucker et al., 2004). There are several approaches to examining creativity within different contexts, including everyday creativity (e.g., Goslin-Jones & Richards, 2018), hence we recommend that future research should examine mindfulness effects on creativity in real-world settings and on a far broader range of tasks than those that have become the mainstay of laboratory-based research with convergent and divergent thinking tasks. We have also noted how the dual-process distinction between Type 1 and Type 2 processes may align with the distinction between idea generation and idea evaluation (cf. Sowden et al., 2015), although the validity of this alignment would benefit from further analysis, as would the specific influence of mindfulness on Type 1 and Type 2 processing in creative situations.

## Conclusion

The current analyses found evidence for theoretical claims that mindfulness enhances creativity, with a small to medium effect size arising for studies using control group designs and a medium effect size arising for studies using pretest–posttest designs. However, discrepant findings were observed for moderator variables. Control group variations significantly influenced the effectiveness of mindfulness on creative performance, although we note our concerns with waiting-list designs possibly artificially inflating effect sizes. Convergent thinking tasks benefitted more from a mindfulness intervention relative to divergent thinking tasks for both analyses, suggesting that mindfulness has a differential effect on the underpinning cognitive processes associated with these tasks. Intervention length significantly affected the success of the intervention in control group studies but did not affect the success of the intervention in pretest–posttest studies, with no clear explanation for this variation. In both meta-analyses, medium length (20 min to 1 week) interventions displayed medium to large effect sizes and were deemed to be the most effective for enhancing creativity.

Based on our findings, we recommend that future research continues to utilize active control groups that involve tasks that match mindfulness interventions in nonspecific factors while also excluding a mindfulness element so that differences between groups can be accurately attributed to the presence versus absence of mindfulness. We propose a theoretical basis for our observed effects that relates to the role of mindfulness in enhancing attentional and WM processes that can then facilitate creativity both on divergent and convergent thinking tasks in specific ways. This explanation makes sense of the benefits that can arise from mindfulness interventions on creative performance, thereby potentially guiding future research in advancing the development of underlying theoretical accounts of the relationship between mindfulness and creativity.

### Supplementary Information

Below is the link to the electronic supplementary material.

Supplementary file1 (DOCX 712 KB)

Supplementary file2 (XLS 46 KB)

Supplementary file3 (ZIP 20 KB)

Supplementary file4 (ZIP 19 KB)

## Data Availability

We adhered to the Meta-Analytic Reporting Standards (MARS) for meta-analytic reporting (Appelbaum et al., 2018). The Preferred Reporting Items for Systematic Reviews and Meta-Analyses (PRISMA) statement was used to guide the review process (Moher et al., 2009). All meta-analytic data and research materials have been made publicly available at The Open Science Framework (https://osf.io/e9ums/?view_only=9d8dadd4017e4a4c9b392539dda473af).

## Code availability

Data were analyzed using Comprehensive Meta-Analysis (CMA; Bornstein et al., 2013) Version 3.0. This review project was not preregistered.

## References

[CR1] Abraham A (2018). The neuroscience of creativity.

[CR2] Acar S, Burnett C, Cabra JF (2017). Ingredients of creativity: Originality and more. Creativity Research Journal.

[CR3] Amabile TM, Barsade SG, Mueller JS, Staw BM (2005). Affect and creativity at work. Administrative Science Quarterly.

[CR4] Anderson ND, Lau MA, Segal ZV, Bishop SR (2007). Mindfulness-based stress reduction and attentional control. Clinical Psychology and Psychotherapy.

[CR5] Anicha CL, Ode S, Moeller SK, Robinson MD (2012). Toward a cognitive view of trait mindfulness: Distinct cognitive skills predict its observing and nonreactivity facets. Journal of Personality.

[CR6] Appelbaum M, Cooper H, Kline RB, Mayo-Wilson E, Nezu AM, Rao SM (2018). Journal article reporting standards for quantitative research in psychology: The APA publications and Communications Board task force report. American Psychologist.

[CR7] Ash IK, Wiley J (2006). The nature of restructuring in insight: An individual-differences approach. Psychonomic Bulletin & Review.

[CR8] Baas M, Nevicka B, ten Velden FS (2014). Specific mindfulness skills differentially predict creative performance. Personality and Social Psychology Bulletin.

[CR9] Baas M, Nevicka B, ten Velden FS (2020). When paying attention pays off: The mindfulness skill act with awareness promotes creative idea generation in groups. European Journal of Work and Organizational Psychology.

[CR10] Baer RA (2003). Mindfulness training as a clinical intervention: A conceptual and empirical review. Clinical Psychology: Science and Practice.

[CR11] Baer RA, Smith GT, Allen KB (2004). Assessment of mindfulness by self-report: The Kentucky inventory of mindfulness skills. Assessment.

[CR12] Baer RA, Smith GT, Hopkins J, Krietemeyer J, Toney L (2006). Using self-report assessment methods to explore facets of mindfulness. Assessment.

[CR13] Bailey, N. W., Freedman, G., Raj, K., Sullivan, C. M., Rogasch, N. C., Chung, S. W., Hoy, K. E., Chambers, R., Hassed, C., van Dam, N. T., Koenig, T., & Fitzgerald, P. B. (2019). Mindfulness meditators show altered distributions of early and late neural activity markers of attention in a response inhibition task. *PLOS ONE*, *14*(8), 1–15. e0203096. 10.1371/journal.pone.0203096 10.1371/journal.pone.0203096 PMC6684080 31386663

[CR14] Baird B, Smallwood J, Mrazek MD, Kam JWY, Franklin MS, Schooler JWP (2012). Inspired by distraction: Mind wandering facilitates creative incubation. Psychological Science.

[CR15] Ball, L. J., & Stevens, A. (2009). Evidence for a verbally-based analytic component to insight problem-solving. In N. A. Taatgen & H. van Rijn (Eds.), *Proceedings of the thirty-first annual conference of the cognitive science society* (pp. 1060–1065)*.* Cognitive Science Society.

[CR16] Ball LJ, Marsh JE, Litchfield D, Cook RL, Booth N (2015). When distraction helps: Evidence that concurrent articulation and irrelevant speech can facilitate insight problem-solving. Thinking and Reasoning.

[CR17] Beaty RE, Silvia PJ (2012). Why do ideas get more creative across time? An executive interpretation of the serial order effect in divergent thinking tasks. Psychology of Aesthetics, Creativity, and the Arts.

[CR18] Beaty RE, Benedek M, Barry Kaufman S, Silvia PJ (2015). Default and executive network coupling supports creative idea production. Scientific Reports.

[CR19] Beaty RE, Benedek M, Silvia PJ, Schacter DL (2016). Creative cognition and brain network dynamics. Trends in Cognitive Sciences.

[CR20] Beaty RE, Kenett YN, Christensen AP, Rosenberg MD, Benedek M, Chen Q, Fink A, Qiu J, Kwapil TR, Kane MJ, Silvia PJ (2018). Robust prediction of individual creative ability from brain functional connectivity. Proceedings of the National Academy of Sciences of the United States of America.

[CR21] Beaty RE, Chen Q, Christensen AP, Kenett YN, Silvia PJ, Benedek M, Schacter DL (2020). Default network contributions to episodic and semantic processing during divergent creative thinking: A representational similarity analysis. NeuroImage.

[CR22] Becerra R, Dandrade C, Harms C (2017). Can specific attentional skills be modified with mindfulness training for novice practitioners?. Current Psychology.

[CR23] Berkovich-Ohana A, Glicksohn J, Ben-Soussan TD, Goldstein A (2017). Creativity is enhanced by long-term mindfulness training and is negatively correlated with trait default-mode-related low-gamma inter-hemispheric connectivity. Mindfulness.

[CR24] Binder JR, Frost JA, Hammeke TA, Bellgowan PSF, Rao SM, Cox RW (1999). Conceptual processing during the conscious resting state: A functional MRI study. Journal of Cognitive Neuroscience.

[CR25] Bishop SR (2002). What do we really know about mindfulness-based stress reduction?. Psychosomatic Medicine.

[CR26] Bornstein MH, Jager J, Putnick DL (2013). Sampling in developmental science: Situations, shortcomings, solutions, and standards. Developmental Review.

[CR27] Bowden EM, Jung-Beeman M, Fleck J, Kounios J (2005). New approaches to demystifying insight. Trends in Cognitive Sciences.

[CR28] Bravo AJ, Pearson MR, Wilson AD, Witkiewitz K (2018). When traits match states: Examining the associations between self-report trait and state mindfulness following a state mindfulness induction. Mindfulness.

[CR29] Brewer JA, Worhunsky PD, Gray JR, Tang YY, Weber J, Kober H (2011). Meditation experience is associated with differences in default mode network activity and connectivity. Proceedings of the National Academy of Sciences of the United States of America.

[CR30] Brown KW, Ryan RM (2003). The benefits of being present: Mindfulness and its role in psychological well-being. Journal of Personality and Social Psychology.

[CR31] Calamia M, Markon K, Tranel D (2012). Scoring higher the second time around: Meta-analyses of practice effects in neuropsychological assessment. Clinical Neuropsychologist.

[CR32] Capurso, V., Fabbro, F., & Crescentini, C. (2014). Mindful creativity: The influence of mindfulness meditation on creative thinking. *Frontiers in Psychology 4*(1), Article 1020, 1–2.10.3389/fpsyg.2013.01020 10.3389/fpsyg.2013.01020 PMC3887545 24454303

[CR33] Carlsson A, Waters N, Waters S, Carlsson ML (2000). Network interactions in schizophrenia - therapeutic implications. Brain Research Reviews.

[CR34] Carmody J, Baer RA (2009). How long does a mindfulness-based stress reduction program need to be? A review of class contact hours and effect sizes for psychological distress. Journal of Clinical Psychology.

[CR35] Carroll JB, Guilford JP (1968). The nature of human intelligence. American Educational Research Journal.

[CR36] Chermahini, S. A., & Hommel, B. (2011). Neural and cognitive mechanisms of creativity. *Cognition*, *115*(3), 1662–5110. Retrieved from https://hdl.handle.net/1887/17977

[CR37] Chiesa A (2013). The difficulty of defining mindfulness: Current thought and critical issues. Mindfulness.

[CR38] Chin, B., Lindsay, E. K., Greco, C. M., Brown, K. W., Smyth, J. M., Wright, A. G. C., & Creswell, J. D. (2021). Mindfulness interventions improve momentary and trait measures of attentional control: Evidence from a randomized controlled trial. *Journal of Experimental Psychology, 150*(4), 686–699. 10.1037/xge0000969 10.1037/xge0000969 PMC9245911 32969686

[CR39] Christensen PR, Guilford JP, Wilson RC (1957). Relations of creative responses to working time and instructions. Journal of Experimental Psychology.

[CR40] Cogdell-Brooke LS, Sowden PT, Violante IR, Thompson HE (2020). A meta-analysis of functional magnetic resonance imaging studies of divergent thinking using activation likelihood estimation. Human Brain Mapping.

[CR41] Cohen J (1992). Quantitative methods in psychology. Psychological Bulletin.

[CR42] Corazza, G. E. (2016). Potential originality and effectiveness: The dynamic definition of creativity. *Creativity Research Journal*, *28*(3), 258–267. 10.1080/10400419.2016.1195627

[CR43] Colzato LS, Ozturk A, Hommel B (2012). Meditate to create: The impact of focused-attention and open-monitoring training on convergent and divergent thinking. Frontiers in Psychology.

[CR44] Colzato LS, Szapora A, Lippelt D, Hommel B (2017). Prior meditation practice modulates performance and strategy use in convergent- and divergent-thinking problems. Mindfulness.

[CR45] Cropley A (2006). In praise of convergent thinking. Creativity Research Journal.

[CR46] Cunningham JA, Kypri K, McCambridge J (2013). Exploratory randomized controlled trial evaluating the impact of a waiting list control design. BMC Medical Research Methodology.

[CR47] Danek AH, Vallée-Tourangeau F (2018). Magic tricks, sudden restructuring, and the Aha! experience: A new model of nonmonotonic problem-solving. Insight: On the origins of new ideas.

[CR48] Davis MA (2009). Understanding the relationship between mood and creativity: A meta-analysis. Organizational Behavior & Human Decision Processes.

[CR49] De Boer MR, Waterlander WE, Kuijper LDJ, Steenhuis IHM, Twisk JWR (2015). Testing for baseline differences in randomized controlled trials: An unhealthy research behavior that is hard to eradicate. International Journal of Behavioral Nutrition and Physical Activity.

[CR50] DeCaro MS, Van Stockum CA, Wieth MB (2016). When higher working memory capacity hinders insight. Journal of Experimental Psychology: Learning Memory and Cognition.

[CR51] de Sousa, G. M., de Lima-Araújo, G. L., de Araújo, D. B., & de Sousa, M. B. C. (2021). Brief mindfulness-based training and mindfulness trait attenuate psychological stress in university students: a randomized controlled trial. *BMC Psychology*, *9*(1), 21, 1–14. 10.1186/s40359-021-00520-x 10.1186/s40359-021-00520-x PMC7852130 33526085

[CR52] Dimitrov DM, Rumrill PD (2003). Pretest–posttest designs and measurement of change. Work.

[CR53] Ding X, Tang YY, Tang R, Posner MI (2014). Improving creativity performance by short-term meditation. Behavioral and Brain Functions.

[CR54] Dix, A., Ormerod, T., Twidale, M., Sas, C., Gomes da Silva, P. A., & McKnight, L. (2006). Why bad ideas are a good idea. *Proceedings of HCIEd 2006, *Ballina/Killaloe, Ireland.

[CR55] Ding X, Tang YY, Cao C, Deng Y, Wang Y, Xin X, Posner MI (2015). Short-term meditation modulates brain activity of insight evoked with solution cue. Social Cognitive and Affective Neuroscience.

[CR56] Ding X, Tang YY, Deng Y, Tang R, Posner MI (2015). Mood and personality predict improvement in creativity due to meditation training. Learning and Individual Differences.

[CR57] Doll A, Hölzel BK, Boucard CC, Wohlschläger AM, Sorg C (2015). Mindfulness is associated with intrinsic functional connectivity between default mode and salience networks. Frontiers in Human Neuroscience.

[CR58] Dunning DL, Griffiths K, Kuyken W, Crane C, Foulkes L, Parker J, Dalgleish T (2019). Research review: The effects of mindfulness-based interventions on cognition and mental health in children and adolescents—a meta-analysis of randomized controlled trials. Journal of Child Psychology and Psychiatry and Allied Disciplines.

[CR59] Duval S, Tweedie R (2000). Trim and fill: A simple funnel-plot-based method of testing and adjusting for publication bias in meta-analysis. Biometrics.

[CR60] Duyx B, Urlings MJE, Swaen GMH, Bouter LM, Zeegers MP (2017). Scientific citations favor positive results: A systematic review and meta-analysis. Journal of Clinical Epidemiology.

[CR61] Ellamil M, Dobson C, Beeman M, Christoff K (2012). Evaluative and generative modes of thought during the creative process. NeuroImage.

[CR62] Evans JStBT, Stanovich KE (2013). Dual-process theories of higher cognition: Advancing the debate. Perspectives on Psychological Science.

[CR63] Fan J, McCandliss BD, Sommer T, Raz A, Posner MI (2002). Testing the efficiency and independence of attentional networks. Journal of Cognitive Neuroscience.

[CR64] Farias M, Wikholm C (2016). Has the science of mindfulness lost its mind?. BJPsych Bulletin.

[CR65] Fischer R, Hommel B (2012). Deep thinking increases task-set shielding and reduces shifting flexibility in dual-task performance. Cognition.

[CR66] Field AP, Gillett R (2010). How to do a meta-analysis. British Journal of Mathematical and Statistical Psychology.

[CR67] Freeman PR, Hedges LV, Olkin I (1986). Statistical methods for meta-analysis. Biometrics.

[CR68] Furukawa TA, Noma H, Caldwell DM, Honyashiki M, Shinohara K, Imai H, Chen P, Hunot V, Churchill R (2014). Waiting list may be a nocebo condition in psychotherapy trials: A contribution from network meta-analysis. Acta Psychiatrica Scandinavica.

[CR69] Garland EL, Howard MO (2013). Mindfulness-oriented recovery enhancement reduces pain attentional bias in chronic pain patients. Psychotherapy & Psychosomatics.

[CR70] Gazzaley A, Cooney JW, Rissman J, D' Esposito, M.  (2005). Top-down suppression deficit underlies working memory impairment in normal aging. Nature Neuroscience.

[CR71] Giancola, M., Palmiero, M., & D’Amico, S. (2022). Divergent but not convergent thinking mediates the trait emotional intelligence-real-world creativity link: An empirical study. *Creativity Research Journal*, 1–9. 10.1080/10400419.2022.2092338

[CR72] Giannandrea A, Simione L, Pescatori B, Ferrell K, Olivetti Belardinelli M, Hickman SD, Raffone A (2019). Effects of the mindfulness-based stress reduction program on mind wandering and dispositional mindfulness facets. Mindfulness.

[CR73] Gilhooly KJ, Ball LJ, Macchi L (2015). Insight and creative thinking processes: Routine and special. Thinking and Reasoning.

[CR74] Giovannoli J, Martella D, Casagrande M (2021). Assessing the three attentional networks and vigilance in the adolescence stages. Brain Sciences.

[CR75] Goldberg SB, Wielgosz J, Dahl C, Schuyler B, MacCoon DS, Rosenkranz M, Lutz A, Sebranek CA, Davidson RJ (2016). Does the Five Facet Mindfulness Questionnaire measure what we think it does? Construct validity evidence from an active controlled randomized clinical trial. Psychological Assessment.

[CR76] Goslin-Jones, T., & Richards, R. (2018). Mysteries of creative process: Explorations at work and in daily life. *The Palgrave handbook of creativity at work* (pp. 71–106). Palgrave.

[CR77] Gouda S, Luong MT, Schmidt S, Bauer J (2016). Students and teachers benefit from mindfulness-based stress reduction in a school-embedded pilot study. Frontiers in Psychology.

[CR78] Goyal M, Singh S, Sibinga EMS, Gould NF, Rowland-Seymour A, Sharma R, Berger Z, Sleicher D, Maron DD, Shihab HM, Ranasinghe PD, Linn S, Saha S, Bass EB, Haythornthwaite JA (2014). Meditation programs for psychological stress and well-being: A systematic review and meta-analysis. JAMA Internal Medicine.

[CR79] Grossman P (2008). On measuring mindfulness in psychosomatic and psychological research. Journal of Psychosomatic Research.

[CR80] Guilford JP (1967). The nature of human intelligence.

[CR81] Haddaway NR, Page MJ, Pritchard CC, McGuinness L, A.  (2022). PRISMA2020: An R package and Shiny app for producing PRISMA 2020-compliant flow diagrams, with interactivity for optimised digital transparency and open synthesis. Zenodo.

[CR82] Harbord RM, Egger M, Sterne JAC (2006). A modified test for small-study effects in meta-analyses of controlled trials with binary endpoints. Statistics in Medicine.

[CR83] Hart T, Bagiella E (2012). Design and implementation of clinical trials in rehabilitation research. Archives of Physical Medicine and Rehabilitation.

[CR84] Hart T, Fann JR, Novack TA (2008). The dilemma of the control condition in experience-based cognitive and behavioural treatment research. Neuropsychological Rehabilitation.

[CR85] Hass RW (2017). Semantic search during divergent thinking. Cognition.

[CR86] Henriksen D, Richardson C, Shack K (2020). Mindfulness and creativity: Implications for thinking and learning. Thinking Skills and Creativity.

[CR87] Hesser H, Weise C, Rief W, Andersson G (2011). The effect of waiting: A meta-analysis of wait-list control groups in trials for tinnitus distress. Journal of Psychosomatic Research.

[CR88] Higgins JPT, Thompson SG, Deeks JJ, Altman DG (2003). Measuring inconsistency in meta-analyses. British Medical Journal.

[CR89] Hommel B (2012). Convergent and divergent operations in cognitive search. Cognitive Search: Evolution, Algorithms, and the Brain.

[CR90] Howarth A, Smith JG, Perkins-Porras L, Ussher M (2019). Effects of brief mindfulness-based interventions on health-related outcomes: A systematic review. Mindfulness.

[CR91] Jedrczak A, Toomey M, Clements G (1986). The TM-Sidhi programme, age, and brief tests of perceptual-motor speed and nonverbal intelligence. Journal of Clinical Psychology.

[CR92] Josefsson T, Larsman P, Broberg AG, Lundh LG (2011). Self-reported mindfulness mediates the relation between meditation experience and psychological well-being. Mindfulness.

[CR93] Justo CF, Mañas IM, Ayala ES (2014). Improving the graphic creativity levels of Latin American high school students currently living in Spain by means of a mindfulness program. Procedia—Social and Behavioral Sciences.

[CR94] Justo, F. C. (2009). Reduction of the perception of stress in teaching students through the practice of flow meditation. *Psychology Notes, 27*(1), 99–109.

[CR95] Kabat-Zinn J (1982). An outpatient program in behavioral medicine for chronic pain patients based on the practice of mindfulness meditation: Theoretical considerations and preliminary results. General Hospital Psychiatry.

[CR96] Kabat-Zinn J (1990). Full catastrophe living: Using the wisdom of your mind and body to face stress, pain, and illness.

[CR97] Kabat-Zinn J (2003). Mindfulness-based interventions in context: Past, present, and future. Clinical Psychology: Science and Practice.

[CR98] Keller AS, Leikauf JE, Holt-Gosselin B, Staveland BR, Williams LM (2019). Paying attention to attention in depression. Translational Psychiatry.

[CR99] Keng SL, Smoski MJ, Robins CJ (2011). Effects of mindfulness on psychological health: A review of empirical studies. Clinical Psychology Review.

[CR100] Khoury B, Sharma M, Rush SE, Fournier C (2015). Mindfulness-based stress reduction for healthy individuals: A meta-analysis. Journal of Psychosomatic Research.

[CR101] Kiken LG, Garland EL, Bluth K, Palsson OS, Gaylord SA (2015). From a state to a trait: Trajectories of state mindfulness in meditation during intervention predict changes in trait mindfulness. Personality and Individual Differences.

[CR102] Kim J, Shin W (2014). How to do random allocation (randomization). Clinics in Orthopedic Surgery.

[CR103] Kounios J, Frymiare JL, Bowden EM, Fleck JI, Subramaniam K, Parrish TB, Jung-Beeman M (2006). The prepared mind: neural activity prior to problem presentation predicts subsequent solution by sudden insight. Psychological Science.

[CR104] Kwak S, Kim SY, Bae D, Hwang WJ, Cho KIK, Lim KO, Park HY, Lee TY, Kwon JS (2020). Enhanced attentional network by short-term intensive meditation. Frontiers in Psychology.

[CR105] Larson MJ, Steffen PR, Primosch M (2013). The impact of a brief mindfulness meditation intervention on cognitive control and error-related performance monitoring. Frontiers in Human Neuroscience.

[CR106] Lebuda I, Zabelina DL, Karwowski M (2016). Mind full of ideas: A meta-analysis of the mindfulness–creativity link. Personality and Individual Differences.

[CR107] Lee CS, Therriault DJ (2013). The cognitive underpinnings of creative thought: A latent variable analysis exploring the roles of intelligence and working memory in three creative thinking processes. Intelligence.

[CR108] Leszczynski M, Chaieb L, Reber TP, Derner M, Axmacher N, Fell J (2017). Mind wandering simultaneously prolongs reactions and promotes creative incubation. Scientific Reports.

[CR109] Li J, Zhang D, Liang A, Liang B, Wang Z, Cai Y, Gao M, Gao Z, Chang S, Jiao B, Huang R, Liu M (2017). High transition frequencies of dynamic functional connectivity states in the creative brain. Scientific Reports.

[CR110] Li Y, Yang N, Zhang Y, Xu W, Cai L (2021). The relationship among trait mindfulness, attention, and working memory in junior school students under different stressful situations. Frontiers in Psychology.

[CR111] Lin L, Chu H (2018). Quantifying publication bias in meta-analysis. Biometrics.

[CR112] Lindsay EK, Chin B, Greco CM, Young S, Brown KW, Wright AGC, Smyth JM, Burkett D, Creswell JD (2018). How mindfulness training promotes positive emotions: Dismantling acceptance skills training in two randomized controlled trials. Journal of Personality and Social Psychology.

[CR113] Lipsey, M. W., Wilson, D. B. (2001). Practical meta-analysis. *Applied Social Research Methods Series*, *49.*

[CR114] Lueke A, Lueke N (2019). Mindfulness improves verbal learning and memory through enhanced encoding. Memory & Cognition.

[CR115] MacCoon DG, Imel ZE, Rosenkranz MA, Sheftel JG, Weng HY, Sullivan JC, Bonus KA, Stoney CM, Salomons TV, Davidson RJ, Lutz A (2012). The validation of an active control intervention for Mindfulness Based Stress Reduction (MBSR). Behaviour Research and Therapy.

[CR116] MacGregor JN, Ormerod TC, Chronicle EP (2001). Information processing and insight: A process model of performance on the nine-dot and related problems. Journal of Experimental Psychology: Learning Memory and Cognition.

[CR117] Malinowski P (2013). Neural mechanisms of attentional control in mindfulness meditation. Frontiers in Neuroscience.

[CR118] Marsh JE, Threadgold E, Barker ME, Litchfield D, Degno F, Ball LJ (2021). The susceptibility of compound remote associate problems to disruption by irrelevant sound: A Window onto the component processes underpinning creative cognition?. Journal of Cognitive Psychology.

[CR119] Masicampo EJ, Baumeister RF (2007). Relating mindfulness and self-regulatory processes. Psychological Inquiry.

[CR120] Matko K, Sedlmeier P (2019). What is meditation? Proposing an empirically derived classification system. Frontiers in Psychology.

[CR121] McCarney R, Warner J, Iliffe S, van Haselen R, Griffin M, Fisher P (2007). The Hawthorne effect: A randomised, controlled trial. BMC Medical Research Methodology.

[CR122] Mrazek, M. D., Franklin, M. S., Phillips, D. T., Baird, B., & Schooler, J. W. (2013). Mindfulness training improves working memory capacity and GRE performance while reducing mind wandering. *Psychological Science, 24*(5), 776–781. 10.1177/0956797612459659 10.1177/0956797612459659 23538911

[CR123] Mednick, S. A., & Mednick, M. T. (1967). *Remote Associates Test: College and adult forms 1 and 2* [Examiner’s manual]*.* Houghton Mifflin.

[CR124] Mednick, S. (1962). The associative basis of the creative process. *Psychological Review, 69*(3), 220–232. 10.1037/h0048850 10.1037/h0048850 14472013

[CR125] Mooneyham BW, Schooler JW (2013). The costs and benefits of mind-wandering: A review. Canadian Journal of Experimental Psychology.

[CR126] Moher, D., Liberati, A., Tetzlaff, J., Altman, D. G., & PRISMA Group (2009). Preferred reporting items for systematic reviews and meta-analyses: The PRISMA statement. *PLoS Medicine, 6*(7), 1–6. 10.1371/journal.pmed.1000097 10.1371/journal.pmed.1000097 PMC2707599 19621072

[CR127] Mrazek MD, Smallwood J, Schooler JW (2012). Mindfulness and mind-wandering: Finding convergence through opposing constructs. Emotion.

[CR128] Müller BCN, Gerasimova A, Ritter SM (2016). Concentrative meditation influences creativity by increasing cognitive flexibility. Psychology of Aesthetics, Creativity, and the Arts.

[CR129] Noone C, Hogan MJ (2018). A randomised active-controlled trial to examine the effects of an online mindfulness intervention on executive control, critical thinking and key thinking dispositions in a university student sample. BMC Psychology.

[CR130] Norris CJ, Creem D, Hendler R, Kober H (2018). Brief mindfulness meditation improves attention in novices: Evidence from ERPs and moderation by neuroticism. Frontiers in Human Neuroscience.

[CR131] Oberauer, K. (2019). Working memory and attention: A conceptual analysis and review. *Journal of Cognition*, *2*(1), 36, 1–13. 10.5334/joc.58 10.5334/joc.58 PMC6688548 31517246

[CR132] Öllinger M, Jones G, Knoblich G (2014). The dynamics of search, impasse, and representational change provide a coherent explanation of difficulty in the nine-dot problem. Psychological Research.

[CR133] Ormerod TC, MacGregor JN, Chronicle EP (2002). Dynamics and constraints in insight problem-solving. Journal of Experimental Psychology: Learning Memory and Cognition.

[CR134] Ormerod, T. C., MacGregor, J. N., Chronicle, E. P., Dewald A. D., & Chu, Y. (2013). Act first, think later: The presence and absence of inferential planning in problem solving. *Memory and Cognition, 41*, 1096–1108. 10.3758/s13421-013-0318-5 10.3758/s13421-013-0318-5 23589154

[CR135] Ostafin BD, Kassman KT (2012). Stepping out of history: Mindfulness improves insight problem-solving. Consciousness and Cognition.

[CR136] Ovosi JO, Ibrahim MS, Bello-Ovosi BO (2017). Randomized controlled trials: Ethical and scientific issues in the choice of placebo or active control. Annuals of African Medicine.

[CR137] Park T, Reilly-Spong M, Gross CR (2013). Mindfulness: A systematic review of instruments to measure an emergent patient-reported outcome. Quality of Life Research.

[CR138] Payne SJ, Duggan GB (2011). Giving up problem-solving. Memory & Cognition.

[CR139] Plucker JA (1999). Is the proof in the pudding? Reanalyses of Torrance’s (1958 to present) longitudinal data. Creativity Research Journal.

[CR140] Plucker JA, Beghetto RA, Dow GT (2004). Why isn't creativity more important to educational psychologists? Potentials, pitfalls, and future directions in creativity research. Educational Psychologist.

[CR141] Poure, P., (2016). *The impact of mindfulness meditation on students’ creativity* [Honor’s thesis, Butler University]. Undergraduate Honors Thesis Collection, 349. https://digitalcommons.butler.edu/ugtheses/349

[CR142] Preiss DD, Cosmelli D, Karwowski M, Kaufman JC (2017). Mind wandering, creative writing, and the self. The creative self: Effect of beliefs, self-efficacy, mindset, and identity.

[CR143] Rahl HA, Lindsay EK, Pacilio LE, Brown KW, David Creswell J (2017). Brief mindfulness meditation training reduces mind wandering: The critical role of acceptance. Emotion.

[CR144] Remmers C, Topolinski S, Dietrich DE, Michalak J (2015). Impaired intuition in patients with major depressive disorder. British Journal of Clinical Psychology.

[CR145] Ren J, Huang ZH, Luo J, Wei GX, Ying XP, Ding ZG, Wu YB, Luo F (2011). Meditation promotes insightful problem-solving by keeping people in a mindful and alert conscious state. Science China Life Sciences.

[CR146] Ribeiro JD, Franklin JC, Fox KR, Bentley KH, Kleiman EM, Chang BP, Nock MK (2016). Self-injurious thoughts and behaviors as risk factors for future suicide ideation, attempts, and death: A meta-analysis of longitudinal studies. Psychological Medicine.

[CR147] Roberts RP, Addis DR, Jung RE, Vartanian O (2018). A common mode of processing governing divergent thinking and future imagination. The Cambridge handbook of the neuroscience of creativity.

[CR148] Roberts BW, Lejuez C, Krueger RF, Richards JM, Hill PL (2014). What is conscientiousness and how can it be assessed?. Developmental Psychology.

[CR149] Rominger C, Papousek I, Perchtold CM, Benedek M, Weiss EM, Weber B, Schwerdtfeger AR, Eglmaier MTW, Fink A (2020). Functional coupling of brain networks during creative idea generation and elaboration in the figural domain. NeuroImage.

[CR150] Rothstein HR, Sutton AJ, Borenstein M, Rothstein HR, Sutton AJ, Borenstein M (2006). Publication bias in meta-analysis. Publication bias in meta-analysis: Prevention, assessment and adjustments.

[CR151] Rosenkranz MA, Dunne JD, Davidson RJ (2019). The next generation of mindfulness-based intervention research: What have we learned and where are we headed?. Current Opinion in Psychology.

[CR152] Runco MA, Kaufman JC, Sternberg RJ (2012). Divergent thinking, creativity, and ideation. The Cambridge handbook of creativity.

[CR153] Runco MA, Jaeger GJ (2012). The standard definition of creativity. Creativity Research Journal.

[CR154] Ruocco AC, Direkoglu E (2013). Delineating the contributions of sustained attention and working memory to individual differences in mindfulness. Personality and Individual Differences.

[CR155] Salvi, C., Beeman, M., Bikson, M., McKinley, R., & Grafman, J. (2020). TDCS to the right anterior temporal lobe facilitates insight problem-solving. *Scientific Reports, 10*, Article 946. 10.1038/s41598-020-57724-1 10.1038/s41598-020-57724-1 PMC6976642 31969588

[CR156] Schmertz SK, Anderson PL, Robins DL (2009). The relation between self-report mindfulness and performance on tasks of sustained attention. Journal of Psychopathology and Behavioral Assessment.

[CR157] Schöne, B., Gruber, T., Graetz, S., Bernhof, M., & Malinowski, P. (2018). Mindful breath awareness meditation facilitates efficiency gains in brain networks: A steady-state visually evoked potentials study. *Scientific Reports*, *8*, Article 13687. 10.1038/s41598-018-32046-5 10.1038/s41598-018-32046-5 PMC6135840 30209327

[CR158] Schooler JW, Ohlsson S, Brooks K (1993). Thoughts beyond words: When language overshadows insight. Journal of Experimental Psychology: General.

[CR159] Shapiro SL, Carlson LE, Astin JA, Freedman B (2006). Mechanisms of mindfulness. Journal of Clinical Psychology.

[CR160] Shapiro SL, Jazaieri H, Goldin PR (2012). Mindfulness-based stress reduction effects on moral reasoning and decision making. Journal of Positive Psychology.

[CR161] Shen W, Hommel B, Yuan Y, Chang L, Zhang W (2018). Risk-taking and creativity: Convergent, but not divergent thinking is better in low-risk takers. Creativity Research Journal.

[CR162] Simonton DK, Beghetto R, Sriraman B (2017). Big-C versus little-c creativity: Definitions, implications, and inherent educational contradictions. Creative contradictions in education: Creativity theory and action in education.

[CR163] Simonton DK (2018). Defining creativity: Don't we also need to define what is not creative?. The Journal of Creative Behavior.

[CR164] Sio UN, Ormerod TC (2009). Does incubation enhance problem-solving?. A meta-analytic review. Psychological Bulletin.

[CR165] Smallwood J, Schooler JW (2006). The restless mind. Psychological Bulletin.

[CR166] Smith SM, Ward TB, Holyoak KJ, Morrison RG (2012). Cognition and the creation of ideas. The Oxford handbook of thinking and reasoning.

[CR167] Sobel KV, Gerrie MP, Poole BJ, Kane MJ (2007). Individual differences in working memory capacity and visual search: The roles of top-down and bottom-up processing. Psychonomic Bulletin & Review.

[CR168] Sowden PT, Pringle A, Gabora L (2015). The shifting sands of creative thinking: Connections to dual-process theory. Thinking and Reasoning.

[CR169] Stock-Homburg, R. M., Heald, S. L., Holthaus, C., Gillert, N. L., & von Hippel, E. (2021). Need-solution pair recognition by household sector individuals: Evidence, and a cognitive mechanism explanation. *Research Policy*, *50*(8), 104068, 1–17 10.1016/j.respol.2020.104068

[CR170] Stuyck, H., Cleeremans, A., & Van den Bussche, E. (2022). Aha! Under pressure: The Aha! experience is not constrained by cognitive load. *Cognition*, *219*(7), 104946, 1–15. 10.1016/j.cognition.2021.104946 10.1016/j.cognition.2021.104946 34891110

[CR171] Takeuchi H, Taki Y, Matsudaira I, Ikeda S, Kelssy KH, Nouchi R, Sakaki K, Nakagawa S, Nozawa T, Yokota S, Araki T, Hanawa S, Ishibashi R, Yamazaki S, Kawashima R (2020). Convergent creative thinking performance is associated with white matter structures: Evidence from a large sample study. NeuroImage.

[CR172] Tang YY, Yang L, Leve LD, Harold GT (2012). Improving executive function and its neurobiological mechanisms through a mindfulness-based intervention: Advances within the field of developmental neuroscience. Child Development Perspectives.

[CR173] Tang YY, Hölzel BK, Posner MI (2015). The neuroscience of mindfulness meditation. Nature Reviews Neuroscience.

[CR174] Taren AA, Gianaros PJ, Greco CM, Lindsay EK, Fairgrieve A, Brown KW, Rosen RK, Ferris JL, Julson E, Marsland AL, Creswell JD (2017). Mindfulness meditation training and executive control network resting state functional connectivity: A randomized controlled trial. Psychosomatic Medicine.

[CR175] Thompson C, Quigley E, Taylor A (2021). The influence of a short-term mindfulness meditation intervention on emotion and visual attention. Journal of Cognitive Enhancement.

[CR176] Threadgold E, Marsh JE, Ball LJ (2018). Normative data for 84 UK English rebus puzzles. Frontiers in Psychology.

[CR177] Torrance EP (1972). Can we teach children to think creatively?. The Journal of Creative Behavior.

[CR178] Torrance, E. P. (1984). The role of creativity in identification of the gifted and talented. *Gifted Child Quarterly, 28*(4), 153–156. 10.1177/001698628402800403

[CR179] Van Stockum CA, DeCaro MS (2020). When working memory mechanisms compete: Predicting cognitive flexibility versus mental set. Cognition.

[CR180] Walsh, M. (2013). *Mindfulness and creative performance: Effects of brief and sham mindfulness meditation on insight problem-solving*. Undergraduate Honors Thesis, Dún Laoghaire Institute of Art, Design and Technology, Dún Laoghaire, Ireland.

[CR181] Webb ME, Little DR, Cropper SJ, Roze K (2017). The contributions of convergent thinking, divergent thinking, and schizotypy to solving insight and noninsight problems. Thinking and Reasoning.

[CR182] Weisberg RW (2015). Toward an integrated theory of insight in problem-solving. Thinking and Reasoning.

[CR183] Weisberg RW (2018). Response to Harrington on the definition of creativity. Creativity Research Journal.

[CR184] Wimmer L, Bellingrath S, von Stockhausen L (2020). Mindfulness training for improving attention regulation in university students: Is it effective? And do yoga and homework matter?. Frontiers in Psychology.

[CR185] Wisniewski EJ (1997). When concepts combine. Psychonomic Bulletin and Review.

[CR186] Wolkin JR (2015). Cultivating multiple aspects of attention through mindfulness meditation accounts for psychological well-being through decreased rumination. Psychology Research and Behavior Management.

[CR187] Xu M, Purdon C, Seli P, Smilek D (2017). Mindfulness and mind wandering: The protective effects of brief meditation in anxious individuals. Consciousness and Cognition.

[CR188] Yamaoka, A., & Yukawa, S. (2020). Mind wandering in creative problem-solving: Relationships with divergent thinking and mental health. *PLOS ONE*, *15*(4), Article e0231946, 1–11. 10.1371/journal.pone.0231946 10.1371/journal.pone.0231946 PMC7180068 32325483

[CR189] Zedelius CM, Schooler JW (2015). Mind wandering “Ahas” versus mindful reasoning: Alternative routes to creative solutions. Frontiers in Psychology.

[CR190] Zeng, L., Proctor, R. W., & Salvendy, G. (2011). Can traditional divergent thinking tests be trusted in measuring and predicting real-world creativity? *Creativity Research Journal*, *23*(1), 24–37. https://psycnet.apa.org/doi/10.1080/10400419.2011.545713

[CR191] Zhang Z, Zhang W, Wu X, Tan T, Luo J (2019). Incubation optimizes the promoting effects of rewards on creativity. PsyCh Journal.

[CR192] Zabelina, D. L, Robinson, M. D., Ostafin, B. D., & Council, J. R. (2011). Manipulating mindfulness benefits creative elaboration at high levels of neuroticism. *Empirical Studies of the Arts, 29*(2), 243–255. 10.2190/EM.29.2.g

