# Supplementary material for: A meta-analytical review of the impact of mindfulness on creativity: Framing current lines of research and defining moderator variables
Source: Psychon Bull Rev. 2023 Jul 13;30(6):2155–86. doi: 10.3758/s13423-023-02327-w (PMC10728263; doi:10.3758/s13423-023-02327-w)
Supplement: Supplementary file 1 — Supplementary file1 (DOCX 712 KB) [file 13423_2023_2327_MOESM1_ESM.docx]

**Appendix A**

Summary of Inclusion Criteria

| Inclusion Criteria | Description |
| --- | --- |
| Effect size | All studies report information that allows for the computation of an effect size. |
| Mindfulness intervention | All studies include a mindfulness intervention. These can vary in size as mindfulness intensity/duration will be a moderator variable. |
| Creativity task | All studies include a creativity task rather than just self-report measures. These can vary as divergent/convergent creativity tasks will be a moderator variable. |
| Control group (first meta-analysis only) | All studies include the presence of a control group (i.e., active control, waiting list control or no treatment control). |

**Appendix B**

PRISMA Flowchart of Meta-Analysis Study Inclusion/Exclusion Process


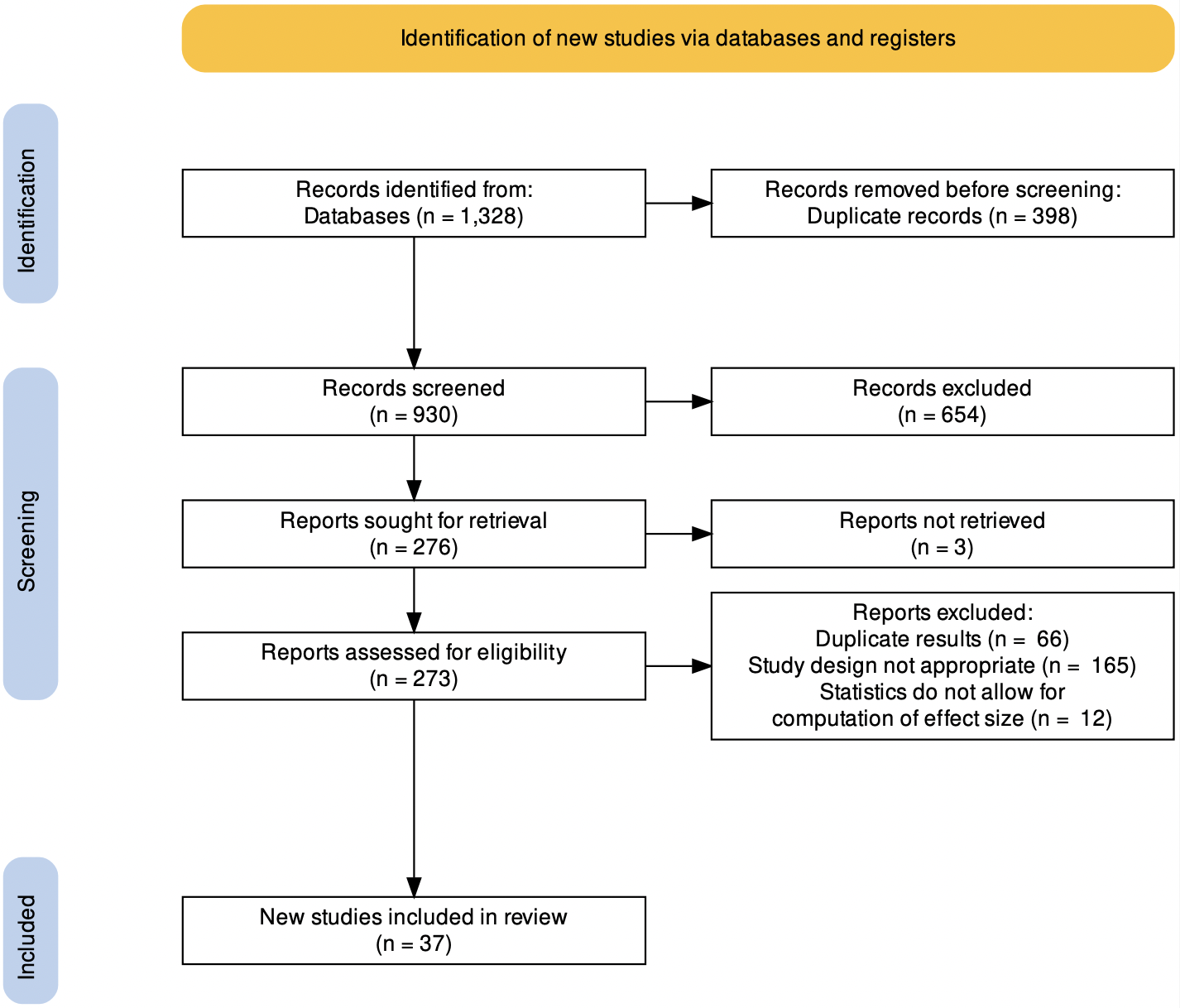


*Note:* Searches were conducted with the following key words and descriptors: mindfulness, and creativ*, intersected with one of convergent, divergent, intervention, MBSR, insight or problem. Overlap in exclusion reasons are possible. Flowchart was created using ‘DiagrammeR’ R package to develop a customisable flow diagram that conforms to PRISMA 2022 standards (Haddaway et al., 2022).

**Appendix C**

Funnel Plot for Meta-Analysis of Control Group Studies

**Appendix D**

Funnel Plot for Meta-Analysis of Pretest-Posttest Studies

**Appendix E**

Risk of Bias Assessment Items with Descriptions and Scoring Methods

| *Item* | *Description* | *Score* |
| --- | --- | --- |
| 1. Quality of reporting | Each reviewer will determine the quality of reporting of the statistics. | 1 = Statistical methods examined changes in outcome measures from: (a) before and after intervention; and/or (b) between control and intervention groups. Analyses reported *p*-values for changes.  2 = Statistical methods examined changes in outcome measures from: (a) before and after intervention; and/or (b) between control and intervention groups. Analyses did not report *p*-values for changes.  3 = Statistical methods did not consider changes before and after intervention and/or between control and intervention groups. |
| 1. Mindfulness intervention | Each reviewer will report on the level of detail provided about the mindfulness intervention utilised in the study. | 1 = Mindfulness intervention clearly described.  2 = Mindfulness intervention described with some missing details.  3 = Intervention not clearly described. |
| 1. Appropriateness of statistical test | Each reviewer will determine whether appropriate statistical tests have been utilised. | 1 = Appropriate tests used.  2 = Not enough information to conclude.  3 = Inappropriate tests used. |

**Appendix E** (continued)

| 1. Similar groups at baseline | Groups are similar on prognostic indicators at baseline (and this was explicitly assessed) or adjustments were made to correct for baseline imbalance. | 1 = Yes.  2 = Unclear.  3 = No. |
| --- | --- | --- |
| 1. Participant withdrawal | Each reviewer will determine the number of participants who withdrew from the intervention. | 1 = Studies with less than or equal to 5% dropouts.  2 = Not stated.  3 = More than 5% dropouts. |
| 1. Randomisation | Each reviewer will determine if the randomisation sequence allows each study participant to have an equal chance of receiving the intervention or being in the control condition (e.g., computer generated random numbers). | 1 = Adequate randomisation.  2 = Unclear “random allocation”.  3 = Non-randomised. |
| 1. Mindfulness measure | Each reviewer will report how many measures of mindfulness are included in each study and list the measures utilised. | 1 = More than one measure of mindfulness is included.  2 = One measure of mindfulness is included.  3 = No measure of mindfulness included. |
| 1. Study question/objectives | Each reviewer will determine if the study question and/or objective is clearly stated. | 1 = Clearly stated.  2 = Unclear.  3 = Not stated. |
| 1. Sample size | Each reviewer will determine if the sample size is based on an adequate power analysis. | 1 = Yes, power 0.8 or above.  2 = Yes, power less than 0.8.  3 = Not stated. |

**Appendix F**

Quality Assessment Scoring of Studies Utilising Control Groups

| Study | 1 | 2 | 3 | 4 | 5 | 6 | 7 | 8 | 9 |
| --- | --- | --- | --- | --- | --- | --- | --- | --- | --- |
| Zabelina et al. (2011) | 1 –Intervention vs. control design and  *p*-values provided | 1 – Intervention clearly  described | 1 – Correlational analyses | 2 – Groups similar in terms of gender split but no official analysis to assess baseline group differences | 2 – Participant withdrawal rates not stated | 1 – Randomisation procedure utilised | 2 –  One measure of mindfulness included | 1 – Study aims clearly stated | 3 – Power analysis not conducted |
| Ren et al. (2011) | 1 – Pretest vs. posttest and intervention vs. control and *p*-values provided | 1 – Intervention clearly described | 2 – ANOVA and  non-parametric analyses used, which reduces study power | 2 – Groups similar in terms of age and gender – no official analysis to assess baseline group differences | 2 – Participant withdrawal rates not stated | 1– Randomisation procedure utilised | 3 –  No mindfulness measures included | 1 – Study aims clearly stated | 3 – Power analysis not conducted |
| Ostafin and Kassman (2012; study 2 sample a) | 1 – Intervention vs. control and pretest vs. posttest and *p*-values reported | 1 – Intervention  clearly described | 1 – ANOVA | 2 – Gender splits equally across groups but no official analysis to assess baseline group differences | 2 – Participant withdrawal rates not stated | 1 – Randomisation procedure utilised | 1 – More than one measure of mindfulness included | 1 – Study aims clearly stated | 3 – Power analysis not conducted |
| Ostafin and Kassman (2012; study 2 sample b) | 1 – Pretest vs. posttest and intervention vs. control and *p*-values reported | 1 – Intervention clearly described | 1 – ANOVA | 2 – Gender split equally across groups but no official analysis to assess baseline group differences | 2 – Participant withdrawal rates not stated | 1 – Randomisation procedure utilised | 1 – More than one measure of mindfulness included | 1 – Study aims clearly stated | 3 – Power analysis not conducted |
| Colzato and Hommel (2012; sample a) | 1 – Pretest vs. posttest and intervention vs. control and *p*-values reported | 1 – Intervention clearly described | 1 – ANOVA | 2 – Groups split by prior meditation experience but no official analysis to assess baseline group differences | 2 – Participant withdrawal rates not stated | 1 – Randomisation procedure utilised | 3 –  No mindfulness measures included | 1 – Study aims clearly stated | 3 – Power analysis not conducted |
| Colzato and Hommel (2012; sample b) | 1 – Pretest vs. posttest and intervention vs. control and *p*-values reported | 1 – Intervention clearly described | 1 - ANOVA | Groups split by prior meditation experience but no official analysis to assess baseline group differences | 2 – Participant withdrawal rates not stated | 1 – Randomisation procedure utilised | 3 –  No mindfulness measures included | 1 – Study aims clearly stated | 3 – Power analysis not conducted |
| Walsh (2013) | 1 – Intervention vs. control and *p*-values reported | 1 – Intervention clearly described | 1 – ANOVA and *t*-tests | 2 – No official analysis to assess baseline group differences; gender appears off-balanced across groups | 2 – Participant withdrawal rates not stated | 1 – Randomisation procedure utilised | 2 – One measure of mindfulness included | 1 – Study aims clearly stated | 3 – Power analysis not conducted |
| Baas et al. (2014) (Study 3) | 1 – Intervention vs. control and *p*-values reported | 1 – Intervention clearly described | 1 – ANOVA and *t*-tests | 2 – Similar educational and demographic backgrounds across groups but no official analysis to assess baseline group differences | 2 –10 participants withdrew (11.9%) | 2 – Participants volunteered for intervention group | 2 – One measure of mindfulness included | 1 – Study aims clearly stated | 3 – Power analysis not conducted |
| Justo et al. (2014; sample a) | 1 – Intervention vs. control *p*-values reported | 1 – Intervention clearly described | 1 – Correlational analyses | 1 – Mann-Whitney test used to assess group differences | 2 – Participant withdrawal rates not stated | 1 – Randomisation procedure utilised | 3 –  No mindfulness measures included | 1 – Study aims clearly stated | 3 – Power analysis not conducted |
| Justo et al. (2014; sample b) | 1 – Intervention vs. control *p*-values reported | 1 – Intervention clearly described | 1 – Correlational analyses | 1 – Mann-Whitney test used to assess group differences | 2 – Participant withdrawal rates not stated | 1 – Randomisation procedure utilised | 3 –  No mindfulness measures included | 1 – Study aims clearly stated | 3 – Power analysis not conducted |
| Ding et al. (2014) | 1 – Intervention vs. control and *p*-values reported | 1 – Intervention clearly described | 1 – ANOVA, *t*-tests and regression | 1 – No difference in TTCT between groups before training | 2 – Participant withdrawal rates not stated | 1 – Randomisation procedure utilised | 3 –  No mindfulness measures included | 1 – Study aims clearly stated | 3 – Power analysis not conducted |
| Colzato et al. (2017) | 1 – Intervention vs. control and *p*-values reported | 1 – Intervention clearly described | 1 – ANOVA and *t*-tests | 2 – No official analysis to assess baseline group differences | 2 – Participant withdrawal rates not stated. | 1 – Randomisation procedure utilised | 3 –  No mindfulness measures included | 1 – Study aims clearly stated | 3 – Power analysis not conducted |
| Ding et al. (2015) | 1 – Intervention vs. control and *p*-values reported | 1 – Intervention clearly described | 1 – ANOVA, *t*-tests and regression | 1 – No differences between groups at baseline found | 2 – Participant withdrawal rates not stated | 1 – Randomisation procedure utilised | 3 –  No mindfulness measures included | 1 – Study aims clearly stated | 3 – Power analysis not conducted |
| Gouda et al. (2016; a) | 1 – Intervention vs. control and *p*-values reported | 1 – Intervention clearly described | 1 – ANCOVA | 1 – No differences between groups at baseline found | 2 – Participant withdrawal rates not stated | 2 – Groups allocated based on schedule and time preference | 1 – More than one measure of mindfulness included | 1 – Study aims clearly stated | 3 – Power analysis not conducted |
| Gouda et al. (2016; b) | 1 – Intervention vs. control and *p*-values reported | 1 – Intervention clearly described | 1 – ANCOVA | 1 – No differences between groups at baseline found | 2 – Participant withdrawal rates not stated | 2 – Groups allocated based on schedule and time preference | 1 – More than one measure of mindfulness included | 1 – Study aims clearly stated | 3 – Power analysis not conducted |
| Muller and Ritter (2016) | 1 – Intervention vs. control and *p*-values reported | 1 – Intervention clearly described | 1 – ANOVA | 1 – Groups matched based on prior meditation experience | 1 – No participant withdrawals | 2 – Groups allocated based on what they usually practice | 3 –  No mindfulness measures included | 1 – Study aims clearly stated | 3 – Power analysis not conducted |
| Poure (2016; sample a) | 1 – Intervention vs. control and *p*-values reported | 1 – Intervention clearly described | 1 – ANOVA | 2 – No official analysis to assess baseline group differences | 2 – Participant withdrawal rates not stated | 1 – Randomisation procedure utilised | 3 –  No mindfulness measures included | 1 – Study aims clearly stated | 3 – Power analysis not conducted |
| Poure (2016; sample b) | 1 – Intervention vs. control and *p*-values reported | 1 – Intervention clearly described | 2 – Six ANOVA’s which inflates error | 2 – No official analysis to assess baseline group differences | 2 – Participant withdrawal rates not stated | 1 – Randomisation procedure utilised | 3 –  No mindfulness measures included | 1 – Study aims clearly stated | 3 – Power analysis not conducted |
| Poure (2016; sample c) | 1 – Intervention vs. control and *p*-values reported | 1 – Intervention clearly described | 2 - Six ANOVA’s which inflates error | 2 – No official analysis to assess baseline group differences | 2 – Participant withdrawal rates not stated | 1 – Randomisation procedure utilised | 3 –  No mindfulness measures included | 1 – Study aims clearly stated | 3 – Power analysis not conducted |
| Baas et al. (2020)  (Study 2) | 1 – Intervention vs. control and *p*-values reported | 1 – Intervention clearly described | 2 – Six ANOVAs which inflates errors | 2 – No official analysis to assess baseline group differences | 1 – Six participants excluded due to missing data (<5%) | 1 – Randomisation procedure utilised | 2 – Multiple measures used to assess specific indices of mindfulness but none directly | 1 – Study aims clearly stated | 1 – Power analysis completed and power of 0.8 |

*Note.* Where necessary, studies that report more than one experiment have been separated into samples. For Gouda et al. (2016), sample a represents the teacher population, and sample b represents the student population. For Poure (2016), sample a represents the RAT as the creative measurement, sample b represents the AUT as the creative measurement and sample c represents the RWT as the creative measurement. For Ostafin and Kassman (2012), sample a represents insight problems as the creative measurement and sample b represents non-insight problems as the creative measurement. For Colzato and Hommel (2012) sample a represents the AUT as the creative measurement and sample b represents the RAT as the creative measurement. For Justo (2014) sample a represents the TTCT as the creative measurement and sample b represents the AUT as the creative measurement.

**Appendix G**

Quality Assessment Scoring of Studies Utilising Pretest-Posttest Designs

| Study | 1 | 2 | 3 | 4 | 5 | 6 | 7 | 8 | 9 |
| --- | --- | --- | --- | --- | --- | --- | --- | --- | --- |
| Franco and Justo (2009) | 1 – Pretest vs. posttest design and *p*-values reported | 1- Intervention clearly described | 2 – *t*-tests only | 1 - No official analysis to assess baseline group differences | 2 – Participant withdrawal rates not stated | 1 – Randomisation procedure utilised | 3 –  No mindfulness measures included | 1 – Study aims clearly stated | 3 – Power analysis not conducted |
| Ren et al. (2011) | 1 – Pretest vs. posttest design and *p*-values reported | 1 – Intervention clearly described | 1 – ANOVA | 2 – Similar age and gender but no official analysis to assess baseline group differences | 2 – Participant withdrawal rates not stated | 1 – Randomisation procedure utilised | 3 –  No mindfulness measures included | 1 – Study aims clearly stated | 3 – Power analysis not conducted |
| Ostafin and Kassman (2012; sample a) | 1 – Pretest vs. posttest design and *p*-values reported | 1 – Intervention clearly described | 1 – ANOVA | 2 – Gender split equally but no official analysis to assess baseline group differences | 2 – Participant withdrawal rates not stated | 1 – Randomisation procedure utilised | 1 – More than one mindfulness measure included | 1 – Study aims clearly stated | 3 – Power analysis not conducted |
| Ostafin and Kassman (2012; sample b) | 1 – Pretest vs. posttest design and *p*-values reported | 1 – Intervention clearly described | 1 – ANOVA | 2 – Gender split equally no official analysis to assess baseline group differences | 2 – Participant withdrawal rates not stated | 1 – Randomisation procedure utilised | 1 – More than one mindfulness measure included | 1 – Study aims clearly stated | 3 – Power analysis not conducted |
| Shapiro et al. (2012) | 1 – Pretest vs. posttest design and *p*-values reported | 1 – Intervention clearly described | 1 – ANOVA | 2 – No official analysis to assess baseline group differences | 1 –  3 participants withdrew (<5%) | 1 – Randomisation procedure utilised | 1 – More than one mindfulness measure included | 1 – Study aims clearly stated | 3 – Power analysis not conducted |
| Walsh (2013) | 1 – Pretest vs. posttest design and *p*-values reported | 1 – Intervention clearly described | 1 – ANOVA and *t*-tests | 2 – No official analysis to assess baseline group differences and gender appears unbalanced | 2 – Participant withdrawal rates not stated | 1 – Randomisation procedure utilised | 2 – One mindfulness measure included | 1 – Study aims clearly stated | 3 – Power analysis not conducted |
| Justo et al. (2014) | 1 – Pretest vs. posttest design and *p*-values reported | 1 – Intervention clearly described | 1 –Correlational analyses | 1 – Mann-Whitney test used to assess group differences | 2 – Participant withdrawal rates not stated | 1 – Randomisation procedure utilised | 3 –  No mindfulness measures included | 1 – Study aims clearly stated | 3 – Power analysis not conducted |
| Baas et al. (2014) | 1 – Pretest vs. posttest design and *p*-values reported | 1 – Intervention clearly described | 1 – ANOVA and *t*-tests | 1 – Similar educational and demographic backgrounds but no official analysis to assess baseline group differences | 3 –  10 participants withdrew (11.9%) | 2 – Participants volunteered for intervention group | 2 – One mindfulness measure included | 1 – Study aims clearly stated | 3 – Power analysis not conducted |
| Ding et al. (2014) | 1 – Pretest vs. posttest design and *p*-values reported | 1 – Intervention clearly described | 1 – ANOVA, *t*-tests and regression | 1 – No difference in TTCT between groups before training | 2 – Participant withdrawal rates not stated | 1 – Randomisation procedure utilised | 3 –  No mindfulness measures included | 1 – Study aims clearly stated | 3 – Power analysis not conducted |
| Tang et al. (2014) | 1 – Pretest vs. posttest design and *p*-values reported | 1 – Intervention clearly described | 1 – ANOVA and *t*-tests | 1 – Groups assessed and are matched | 1 –  1 participant withdrew (<5%) | 1 – Randomisation procedure utilised | 3 –  No mindfulness measures included | 1 – Study aims clearly stated | 3 – Power analysis not conducted |
| Ding et al. (2015) | 1 – Pretest vs. posttest design and *p*-values reported | 1 – Intervention clearly described | 1 – ANOVA, *t*-tests and regression | 1 – Groups assessed and are matched | 2 – Participant withdrawal rates not stated | 1 – Randomisation procedure utilised | 3 –  No mindfulness measures included | 1 – Study aims clearly stated | 3 – Power analysis not conducted |
| Gouda et al. (2016) | 1 – Pretest vs. posttest design and *p*-values reported | 1 – Intervention clearly described | 1 – ANCOVA | 1 – No differences between groups found | 2 – Participant withdrawal rates not stated | 2 – Groups allocated based on schedule and time preference | 1 – More than one mindfulness measure included | 1 – Study aims clearly stated | 3 – Power analysis not conducted |
| Muller et al. (2016) | 1 – Pretest vs. posttest design and *p*-values reported. | 1 – Intervention clearly described | 1 – ANOVA | 1 – Differences in age across groups but matched on prior meditation experience | 2 – Participant withdrawal rates not stated | 2 – Groups allocated based on what they usually practice | 3 –  No mindfulness measures included | 1 – Study aims clearly stated | 3 – Power analysis not conducted |
| Poure (2016; sample c) | 1 – Pretest vs. posttest design and *p*-values reported | 1 – Intervention clearly described | 1 – ANOVA | 2 – No official analysis to assess baseline group differences | 2 – Participant withdrawal rates not stated | 1 – Randomisation procedure utilised | 3 –  No mindfulness measures included | 1 – Study aims clearly stated | 3 – Power analysis not conducted |
| Poure (2016; sample b) | 1 – Pretest vs. posttest design and *p*-values reported | 1 – Intervention clearly described | 1 – ANOVA | 2 – No official analysis to assess baseline group differences | 2 – Participant withdrawal rates not stated | 1 – Randomisation procedure utilised | 3 –  No mindfulness measures included | 1 – Study aims clearly stated | 3 – Power analysis not conducted |
| Poure (2016; sample a) | 1 – Pretest vs. posttest design and *p*-values reported | 1 – Intervention clearly described | 1 – ANOVA | 2 – No official analysis to assess baseline group differences | 2 – Participant withdrawal rates not stated | 1 – Randomisation procedure utilised | 3 –  No mindfulness measures included | 1 – Study aims clearly stated | 3 – Power analysis not conducted |
| Berkovich-Ohana et al. (2016) | 1 – Pretest vs. posttest design and *p*-values reported | 1 – Intervention clearly described | 1 – ANOVA | 1 – Groups matched on age and meditation experience | 2 – Participant withdrawal rates not stated | 2 – Groups allocated based on meditation experience | 3 –  No mindfulness measures included | 1 – Study aims clearly stated | 3 – Power analysis not conducted |

*Note:* Where necessary, studies that report more than one experiment have been separated into samples. For Poure (2016), sample a represents the RAT as the creative measurement, sample b represents the AUT as the creative measurement and sample c represents the RWT as the creative measurement. For Ostafin and Kassman (2012), sample a represents insight problems as the creative measurement and sample b represents non-insight problems as the creative measurement.

**Appendix H**

Additional Information for Publication Bias Analyses of Studies Utilising Control Group Design

**Classic Fail-Safe N**

This meta-analysis incorporates data from 20 studies, which yield a *z*-value of 9.30598 and corresponding 2-tailed *p*-value of < .00001. The fail-safe *N* is 431. This means that we would need to locate and include 431 “null” studies in order for the combined 2-tailed *p*-value to exceed .05. Put another way, there would need to be 21.6 missing studies for every observed study for the effect to be nullified.

**Orwin Fail-Safe N**

The Orwin fail-safe *N* is 65. This means that we would need to locate 65 studies with a mean standard difference in means of 0.2 to bring the combined standard difference in means under 0.25.

**Begg and Mazumdar Rank Correlation Test**

In this case Kendall's tau b (corrected for ties, if any) is 0.00000, with a 1-tailed *p*-value (recommended) of .5 or a 2-tailed *p*-value of 1.0 (based on a continuity-corrected normal approximation).

**Egger's Test of the Intercept**

In this case the intercept (*B0*) is 0.00292, 95% confidence interval (-0.76529, 0.75945), with *t* = 0.00805, *df* = 18. The 1-tailed *p*-value (recommended) is .49683, and the 2-tailed *p*-value is .99367.

**Duval and Tweedie’s Trim and Fill**

Under the fixed effect model the point estimate and 95% confidence interval for the combined studies is 0.41201 (0.36652, 0.45751). Using Trim and Fill these values are unchanged. Under the random effects model the point estimate and 95% confidence interval for the combined studies is 0.41801 (0.29239, 0.54363). Using Trim and Fill these values are unchanged.

**Appendix I**

Additional Information for Publication Bias Analyses of Studies Utilising Pretest-Posttest Design

**Classic Fail-Safe N**

This meta-analysis incorporates data from 17 studies, which yield a *z*-value of 4.37151 and corresponding 2-tailed *p*-value of .00001. The fail-safe *N* is 68. This means that we would need to locate and include 68 “null” studies for the combined 2-tailed *p*-value to exceed .050. Put another way, there would need to be 4.0 missing studies for every observed study for the effect to be nullified.

**Orwin Fail-Safe N**

The Orwin fail-safe *N* is 50. This means that we would need to locate 50 studies with mean standard difference in means of 0.4 to bring the combined standard difference in means under 0.45.

**Begg and Mazumdar Rank Correlation Test**

In this case Kendall's *tau* *b* (corrected for ties, if any) is 0.13235, with a 1-tailed *p*-value (recommended) of 022921 or a 2-tailed *p*-value of .45841 (based on a continuity-corrected normal approximation).

**Egger's Test of the Intercept**

In this case the intercept (*B0*) is -0.05305, 95% confidence interval (-0.98175, 0.87566), with *t* = 0.12174, df = 15. The 1-tailed *p*-value (recommended) is 0.45236, and the 2-tailed *p*-value is 0.90472.

**Duval and Tweedie's Trim and Fill**

Under the fixed effect model the point estimate and 95% confidence interval for the combined studies is 0.59418 (0.37597, 0.81240). Using Trim and Fill these values are unchanged. Under the random effects model the point estimate and 95% confidence interval for the combined studies is 0.59418 (0.37597, 0.81240). Using Trim and Fill these values are unchanged.

**Appendix J**

Random Effects (*d*) with Study Moderator Characteristics for Studies Utilising a Pretest-Posttest Design

| Study Name | Cohen’s *d* | Lower limit | Upper limit | *p* -Value | Categorical moderators |
| --- | --- | --- | --- | --- | --- |
| Walsh (2013) | 0.80 | -1.85 | 3.45 | 0.55 | 1,1 |
| Gouda et al. (2016) | 0.00 | -3.29 | 3.30 | 1.00 | 3,2 |
| Ostafin & Kassman (2012; a) | 0.53 | -0.47 | 1.53 | 0.30 | 1,1 |
| Muller et al. (2016) | 0.15 | -1.68 | 1.98 | 0.87 | 2,2 |
| Justo et al. (2014) | 1.92 | 0.65 | 3.19 | 0.00 | 3,2 |
| Shapiro et al. (2012) | 0.17 | -2.12 | 2.46 | 0.88 | 3,1 |
| Ding et al. (2014; a) | 0.12 | -14.55 | 14.79 | 0.99 | 2,2 |
| Ding et al. (2014; b) | 0.81 | -13.87 | 15.48 | 0.91 | 2,2 |
| Justo (2009) | 1.65 | 0.27 | 3.04 | 0.02 | 3,1 |
| Ren et al. (2011) | 0.28 | -0.97 | 1.52 | 0.66 | 2,1 |
| Baas et al. (2014) | 0.24 | -010 | 1.48 | 0.70 | 3,2 |
| Ding et al. (2015) | 0.82 | 0.38 | 1.26 | 0.00 | 2,2 |
| Berkovich-Ohana et al. (2017) | 0.68 | 0.11 | 1.25 | 0.02 | 1,2 |
| Poure (2016; a) | 0.91 | 0.28 | 1.54 | 0.01 | 1,1 |
| Poure (2016; b) | 0.03 | -0.60 | 0.66 | 0.93 | 1,2 |
| Poure (2016; c) | 0.18 | -0.45 | 0.81 | 0.58 | 1,2 |
| Ostafin & Kassman (2012; b) | 0.19 | -0.08 | 1.19 | 0.72 | 1,2 |
| Overall random effect | 0.59 | 0.38 | 0.81 | 0.00 |  |

*Note:* Categorical moderator variables and codes: Intervention length (1 = under 20 mins, 2 = between 20 mins and 1 week inclusive, 3 = longer than 1 week); Creativity measure (1 = divergent task, 2 = convergent task).

**Appendix K**

Random Effects (*d*) with Study Moderator Characteristics for Studies Utilising a Control Group Design

| Study Name | Cohen’s *d* | Lower limit | Upper limit | *p* -Value | Categorical moderators |
| --- | --- | --- | --- | --- | --- |
| Justo et al. (2014; a) | 1.00 | -0.27 | 2.27 | 0.12 | 3,2,2 |
| Colzato & Hommel (2012; a) | 0.04 | -1.39 | 1.46 | 0.95 | 2,2,1 |
| Baas et al. (2014; a) | 0.24 | 0.15 | 0.33 | 0.00 | 3,2,3 |
| Walsh (2013) | 0.81 | 0.47 | 1.15 | 0.00 | 1,1,1 |
| Gouda et al. (2016a) | 0.20 | -2.60 | 3.00 | 0.89 | 3,2,2 |
| Muller & Ritter (2016) | 0.29 | -1.35 | 1.93 | 0.73 | 2,2,1 |
| Baas et al. (2014; b) | 0.47 | 0.41 | 0.53 | 0.00 | 1,1,1 |
| Justo et al. (2014; b) | 1.28 | 0.01 | 2.55 | 0.05 | 3,2,2 |
| Colzato & Hommel (2012; b) | 0.77 | 0.09 | 1.44 | 0.03 | 2,1,1 |
| Poure (2016; a) | 0.00 | -1.09 | 1.10 | 0.99 | 1,1,1 |
| Poure (2016; b) | 0.03 | -1.23 | 1.29 | 0.96 | 1,2,1 |
| Ostafin & Kassman (2012; b) | 0.19 | -0.05 | 0.43 | 0.11 | 1,1,1 |
| Ding et al. (2015) | 0.82 | 0.08 | 1.56 | 0.03 | 2,2,1 |
| Ding et al. (2014) | 0.17 | -2.16 | 2.49 | 0.89 | 2,2,1 |
| Zabelina et al. (2011) | -0.04 | -1.28 | 1.20 | 0.95 | 1,2,3 |
| Ren et al. (2011) | 0.11 | -0.84 | 1.06 | 0.82 | 2,1,3 |
| Ostafin & Kassman (2012; a) | 0.53 | 0.30 | 0.77 | 0.00 | 1,1,1 |
| Colzato et al. (2017) | 0.10 | -3.21 | 3.41 | 0.95 | 2,1,1 |
| Poure (2016; c) | 0.18 | -0.89 | 1.25 | 0.74 | 1,2,1 |
| Gouda et al. (2016; b) | 0.54 | -2.26 | 3.34 | 0.71 | 3,2,2 |
| Overall random effect | 0.42 | 0.29 | 0.54 | 0.00 |  |

*Note*: Categorical moderator variables and codes: Intervention length (1 = under 20 mins, 2 = between 20 mins and 1 week inclusive, 3 = longer than 1 week); Creativity measure (1 = divergent task, 2 = convergent task); Control group design (1 = active control group, 2 = no-treatment control group, 3 = waiting list control group).

**Appendix L**

Categorical Models for Moderator Variables for Studies Utilising Control Group Designs

|  |  |  |  |  |  | | 95% CI | | |  | | |
| --- | --- | --- | --- | --- | --- | --- | --- | --- | --- | --- | --- | --- |
| Moderator and Levels | *Q* | *df* | *p* | Coefficient (*d*) | | Standard Error | Lower | Upper | *Z*-value | | *p* -Value |  |
| Creativity Measure | 5.87 | 1 | < .001 |  | |  |  |  |  | |  |  |
| Divergent |  |  |  | 0.25 | | 0.06 | 0.16 | 0.34 | -3.27 | | = .001 |  |
| Convergent |  |  |  | 0.47 | | 0.04 | 0.32 | 0.61 | 13.21 | | < .01 |  |
| Intervention Length | 6.20 | 2 | < .001 |  | |  |  |  |  | |  |  |
| Short |  |  |  | 0.44 | | 0.05 | 0.30 | 0.59 | 9.25 | | < .001 |  |
| Medium |  |  |  | 0.55 | | 0.21 | 0.16 | 0.95 | 0.47 | | < .001 |  |
| Long |  |  |  | 0.25 | | 0.09 | 0.16 | 0.34 | -2.21 | | < .001 |  |
| Control Group | 13.93 | 2 | < .001 |  | |  |  |  |  | |  |  |
| Active |  |  |  | 0.47 | | 0.03 | 0.37 | 0.56 | 17.43 | | < .001 |  |
| No-treatment |  |  |  | 0.24 | | 0.42 | 0.15 | 0.33 | 1.3 | | < .001 |  |
| Waiting list |  |  |  | 1.01 | | 0.05 | 0.19 | 1.83 | -4.27 | | < .001 |  |

**Appendix M**

Categorical Models for Moderator Variables for Studies Utilising Pretest-Posttest Designs

|  |  |  |  |  |  | | 95% CI | | |  | | |
| --- | --- | --- | --- | --- | --- | --- | --- | --- | --- | --- | --- | --- |
| Moderator and Levels | *Q* | *df* | *p* | Coefficient (*d*) | | Standard Error | Lower | Upper | *Z*-value | | *p* -Value |  |
| Creativity Measure | 1.04 | 1 | = .53 |  | |  |  |  |  | |  |  |
| Divergent |  |  |  | 0.52 | | 0.23 | 0.24 | 0.80 | 3.5 | | < .001 |  |
| Convergent |  |  |  | 0.80 | | 0.26 | 0.35 | 1.20 | -1.02 | | < .001 |  |
| Intervention Length | 2.64 | 2 | = .27 |  | |  |  |  |  | |  |  |
| Short |  |  |  | 0.45 | | 0.14 | 0.17 | 0.73 | 3.15 | | = .002 |  |
| Medium |  |  |  | 0.73 | | 0.25 | 0.33 | 1.14 | 1.13 | | < .001 |  |
| Long |  |  |  | 1.06 | | 0.38 | 0.23 | 1.87 | 1.64 | | = .002 |  |
